# Carbon-Based Oxamate Cobalt(III) Complexes as Bioenzyme Mimics for Contaminant Elimination in High Backgrounds of Complicated Constituents

**DOI:** 10.3390/ma10101169

**Published:** 2017-10-12

**Authors:** Nan Li, Yun Zheng, Xuemei Jiang, Ran Zhang, Kemei Pei, Wenxing Chen

**Affiliations:** 1 National Engineering Lab for Textile Fiber Materials & Processing Technology (Zhejiang), Zhejiang Sci-Tech University, Hangzhou 310018, China; sylviazy1995@sina.com (Y.Z.); jiangxm1462@hotmail.com (X.J.); zhangran325@126.com (R.Z.); 2 Department of Chemistry, Zhejiang Sci-Tech University, Hangzhou 310018, China; xiaopei027@sina.com

**Keywords:** bioenzyme mimic, target detoxification, carbon nanotubes, substrate capture, ligand effect, oxygen-transfer reaction

## Abstract

Complex wastewater with massive components is now a serious environmental issue facing humanity. Selective removal of low-concentration contaminants in mixed constituents holds great promise for increasing water supplies. Bioenzymes like horseradish peroxidase exhibit oxidizing power and selectivity. Here, we manufactured its mimic through immobilizing non-heme oxamate anionic cobalt(III) complex ([Co^III^(opba)]^−^, opba = o-phenylenebis(oxamate)) onto pyridine (Py) modified multiwalled carbon nanotubes ([Co^III^(opba)]^−^-Py-MWCNTs, MWCNTs = multiwalled carbon nanotubes), where MWCNTs captured substrates and Py functioned as the fifth ligand. We chose typical azo dye (C.I. Acid Red 1) and antibiotic (ciprofloxacin) as model substrates. Without •OH, this catalyst could detoxify target micropollutants efficiently at pH from 8 to 11. It also remained efficient in repetitive tests, and the final products were non-poisonous OH-containing acids. Combined with radical scavenger tests and electron paramagnetic resonance result, we speculated that high-valent cobalt-oxo active species and oxygen atom transfer reaction dominated in the reaction pathway. According to density functional theory calculations, the electron spin density distribution order showed that electron-withdrawing ligand was beneficial for inward pulling the excess electron and lowering the corresponding energy levels, achieving an electrophilic-attack enhancement of the catalyst. With target removal property and recyclability, this catalyst is prospective in water detoxication.

## 1. Introduction

Thousands of industrial and agriculture chemical compounds are being discharged into freshwater system without effective treatment [1,2]. Most toxic pollutants featured with stubborn conjugated structure appear at trace concentrations such as dyes, antibiotics and pesticides, but many of them cause pathogenic concerns due to their chemical durability and bioaccumulation [3,4,5]. The abundance of background inorganic or/and organic constituents make them difficult to deal with [1]. Now the inadequate access to clean water and sanitation is afflicting billions of people throughout the world, and millions of them die annually from pollutant transmission through impure water. Detoxification of wastewaters is considered as one of the most inexpensive and effective campaigns to protect public health and save lives [6]. One of the challenges should be the achievement of targeted hazardous component detoxification to guarantee treatment efficiency [7].

In nature, a variety of crude enzymes exhibit unexpected catalytic activities in converting compounds. For example, cytochrome P450 indispensable to demic metabolism is strong enough to oxidize 100 kcal·mol^−1^ C–H bond through activating dioxygen even though C–H bond is inerter than its own surrounding protein framework [8,9]. Its thiolate-ligated iron(IV)oxo (Fe^IV^=O) active species has been considered as an important model for the study of modern catalysis [10]. Like P450, horseradish peroxidase (HRP), which is contained in the root of a hardy perennial herb, utilizes H_2_O_2_ to oxidize a wide variety of organic and inorganic compounds with a single protoporphyrin IX heme group contained in the protein framework. The imidazole coordinated iron atom binds H_2_O_2_ and forms a ferryl oxo active intermediate [11,12]. Both these two bioenzymes showed eminent chemical activity, but in the absence of the ligand and protein framework, the activity of active centers drastically dropped off [13]. If we introduce fifth ligand and one support to artificial homogeneous catalyst, should its function mechanism offer an arena for the chemist to develop much-needed bio-mimic enzymes?

Over the past few years, researchers have dedicated efforts to mimic natural enzymes with stable and efficient alternatives such as carbon materials and polymer assemblies for different applications [14,15,16,17]. For active entity mimics, the use of transition-metal complexes as catalytic entity has received increasing attention during the last decades and a variety of catalytic systems are available like Fe, Mn, Cr, Pd complexes [18,19,20,21,22,23]. While it is worth noting that the homogeneous active entities of bioenzymes have been studied extensively, the roles that the framework and the fifth ligand play have not been sufficiently studied.

Carbon materials, such as graphene, carbon nanotubes, etc., have garnered much attention due to their excellent properties such as high specific surface area, porosity and excellent conductivity [24,25,26,27]. Considering that multiwalled carbon nanotubes (MWCNTs) possesses good electron conductivity and substrate adsorption ability [28], we introduced MWCNTs as the catalyst support to play the protein framework role of bioenzyme. Cofactors (e.g., the axil ligands) could determine the reacting channels to help produce high oxidation state complexes and to control the activation of oxidants [29,30]. Herein, we established a heterogeneous catalyst through fixing the non-heme active entity, square-planar oxamate anionic cobalt(III) complexes ([Co^III^(opba)]^−^, opba = o-phenylenebis(oxamate)), to MWCNTs via the linkage of pyridine (Py) to mimic the structure and function of naturally occurring enzymes [31]. A typical azo dye, Acid Red 1 (AR1), and one antibiotic, ciprofloxacin (CIP) were chosen as model substrates. The catalyst’s target removal of trace pollutants in water was studied, particularly its performance in the presence of industrial auxiliaries was emphasized. In-situ regeneration and repetitive experiments tested its stability, and the ligand effect was expounded by substrate removal tests and density function theory (DFT) calculations. In conclusion, this catalyst could proceed under alkaline conditions under mild conditions with good recyclability. •OH scavenger experiment, results of electron paramagnetic resonance (EPR) and liquid chromatography–mass spectrometry (LC–MS) claimed the reaction mechanisms and the pathway for oxygen atom transfer reaction.

## 2. Results and Discussion

### 2.1. Characterization

As the content of active entity on MWCNTs is directly related to catalytic activity, TGA and atomic absorption spectrometry (AAS) were employed to detect the loading amount of [Co^III^(opba)]^−^. As shown in Figure S1, the weight loss rate of untreated carbon nanotubes was only about 2.0 wt % at 700 °C, while Py-MWCNTs possessed an excess mass loss of 3.50 wt %, accordingly, the molar ratio of the Py to carbon atom (Py/C) was about 1/189 [32]; a redundant 1.80 wt % mass loss of [Co^III^(opba)]^−^-Py-MWCNTs should be attributed to the thermal decomposition of [Co^III^(opba)]^−^. These data indicated that the resulting product contained both Py and [Co^III^(opba)]^−^, and the loading amount of [Co^III^(opba)]^−^ was about 3.30 wt %. According to the data of atomic absorption spectrometry (AAS) (Figure S2), it could be calculated that the content of cobalt was 2.57 mg/L, equal to 3.34 wt % of [Co^III^(opba)]^−^ loaded on the MWCNTs. This result was in line with the data of TGA.

**Figure 1:**
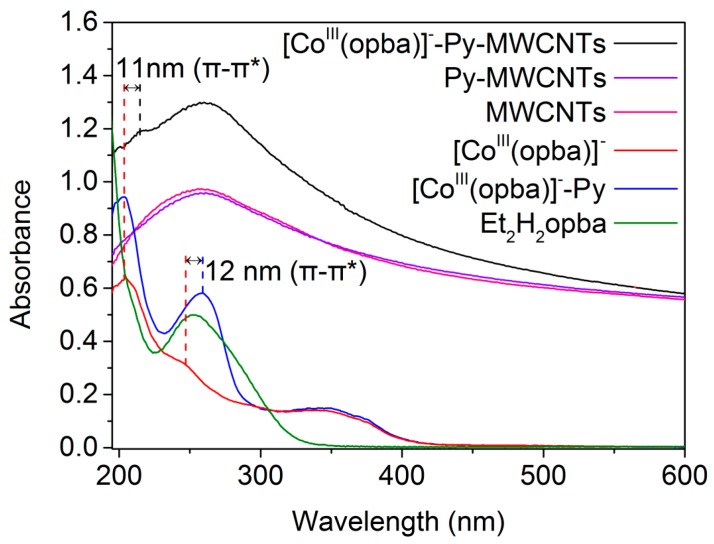
UV-vis adsorption spectra of MWCHTs, Py-MWCNTs, [Co^III^(opba)]^−^-Py-MWCNTs, [Co^III^(opba)]^−^, Py-[Co^III^(opba)]^−^ and Et2H2opba.

To prove whether [Co^III^(opba)]^−^ was physically adsorbed by the supports or chemically combined, UV-vis and X-ray photoelectron spectroscopy (XPS) measurements were carried out. As shown in Figure 1, Py-[Co^III^(opba)]^−^ showed an enhanced adsorption peak over the range 225–300 nm and had a 12 nm red shift compared with [Co^III^(opba)]^−^. This was deduced by the electron-donating effect of Py, because it could increase the conjugation of the complex and decrease the energy level of π–π* transition. This speculation could be proved by the intense adsorption of Et_2_H_2_opba over the range 225–300 nm which was between the range of [Co^III^(opba)]^−^ and Py-[Co^III^(opba)]^−^ [33]. Untreated MWCNTs and Py-MWCNTs showed no adsorption peak near 200 nm, while an adsorption peak of [Co^III^(opba)]^−^-Py-MWCNTs and [Co^III^(opba)]^−^ centered at about 200 nm. Besides, the [Co^III^(opba)]^−^-Py-MWCNTs possessed an 11 nm red shift compared with [Co^III^(opba)]^−^. This indicated that the conjugation of [Co^III^(opba)]^−^ was increased through covalently binding to Py-MWCNTs, and made the inner π–π* transition easier. Therefore, it could be concluded that [Co^III^(opba)]^−^ was successfully grafted onto the MWCNTs via the linkage of Py.

**Figure 2:**
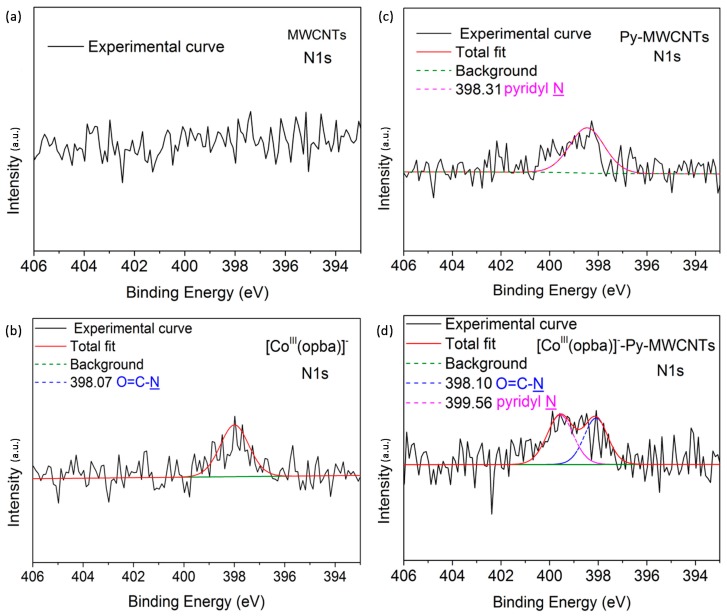
Curve fitting of N1s peaks of (**a**) MWCNTs; (**b**) [Co^III^(opba)]^−^; (**c**) Py-MWCNT and (**d**) [Co^III^(opba)]^−^-Py-MWCNTs.

XPS was employed to further prove the chemical bond type among [Co^III^(opba)]^−^, Py and MWCNTs. According to half-widths of the N1s peaks in Figure 2, we observed that almost no N1s signal was detected in untreated MWCNTs, and the N1s peak of Py-MWCNTs occurred at 398.30 eV originally; after reaction with [Co^III^(opba)]^−^, two N1s peaks centering at 398.10 and 399.56 eV appeared, which were attributed to the N atoms of O=C-N and Py respectively. The distinct 1.25 eV splitting change of Py N may be ascribed to the decreased electron density caused by its electron-donating effect on [Co^III^(opba)]^−^ because of chemical bonding. Notably, the two N1 peak areas were about 1:2, which mean that all the [Co^III^(opba)]^−^ have coordinated with Py because their molecular N ratio is 1:2 too. Therefore, it could be speculated that [Co^III^(opba)]^−^ was grafted onto modified MWCNTs via coordinating with the N atom of Py portion.

In addition, the Co2p levels of the photoelectron spectra are shown in Figure S3. The Co2p_1/2_, Co2p_3/2_ spin–orbit levels of [Co^III^(opba)]^−^ were originally 794.52 and 780.21 eV, after coordinated with Py-MWCNTs, the levels increased to 796.81 and 780.68 eV, respectively. The result was ascribed to the decreased electron density of Co and the increased conjunction of [Co^III^(opba)]^−^ [34]. Moreover, from Figure S4, the C1s spectrum of Py-MWCNTs showed three components at 284.00 eV (caused by MWCNTs), 284.91 eV (caused by C–O) and 286.13 eV (caused by C=O) respectively, and the appearance of C–C=O at 287.61 eV in the spectra of [Co^III^(opba)]^−^-Py-MWCNTs indicated that [Co^III^(opba)]^−^ was chemically linked with Py [35]. These data were consisting with the UV-vis adsorption spectra (Figure 1), in which the increased binding energy implied that [Co^III^(opba)]^−^ had been successfully coordinated to the MWCNTs through the coordination of Co to the N atom of Py.

### 2.2. Catalytic Performance

**Figure 3:**
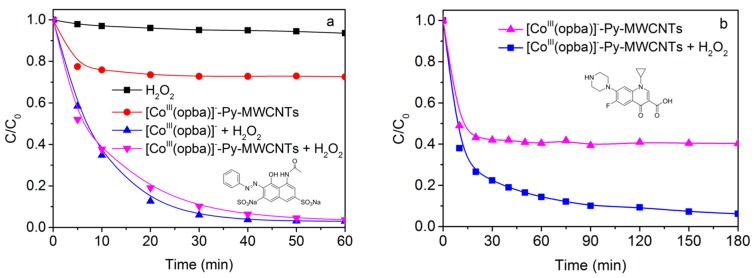
(**a**) Concentration changes of AR1 with 4.0 × 10^−3^ mol/L H_2_O_2_ at 25 °C ([Co^III^(opba)]^−^-Py-MWCNTs = 0.2 g/L, the same mole amount of [Co^III^(opba)]^−^ as [Co^III^(opba)]^−^-Py-MWCNTs, [Co^III^(opba)]^−^ = 0.02 mM, pH 9.0 (0.01 mol/L borate buffer)); and (**b**) CIP with 1.0 × 10^−2^ mol/L H_2_O_2_ at 45 °C. ([Co^III^(opba)]^−^-Py-MWCNTs = 0.2 g/L, pH 8.2 (0.01 mol/L borate buffer)).

AR1 were very stable and hardly decomposed in mere presence of H_2_O_2_ as shown in Figure 3a. When mere [Co^III^(opba)]^−^-Py-MWCNTs were presented, about 25% of AR1 adsorption took place quickly and reached dynamic equilibrium after 20 min. When both [Co^III^(opba)]^−^-Py-MWCNTs and H_2_O_2_ were presented, the concentration of AR1 declined quickly and almost 90% of AR1 was eliminated in 30 min. Under the same conditions, the homogeneous system with [Co^III^(opba)]^−^ (the same mole amount of [Co^III^(opba)]^−^ as [Co^III^(opba)]^−^-Py-MWCNTs) presented similar efficiency for AR1 oxidation. After a lot of experiments, the best experimental conditions were obtained (Figures S5–S7). Although the active centers had smaller encounter probability with substrate in heterogeneous catalysis, overall, there is no significant difference in the activity of the catalyst compared to the unloaded. To investigate its applicability, we also adopted antibiotic CIP as a substrate. In mere presence of H_2_O_2_, CIP is slightly degraded from Figure S8. As shown in Figure 3b, an obvious removal rate disparity appeared between the adsorption and decomposition experiments. Therefore, we concluded that this heterogeneous catalytic system could adsorb substrates, and detoxify them rapidly in the presence of H_2_O_2_ in water.

### 2.3. Effect of High Backgrounds of Complex Constituents

**Figure 4:**
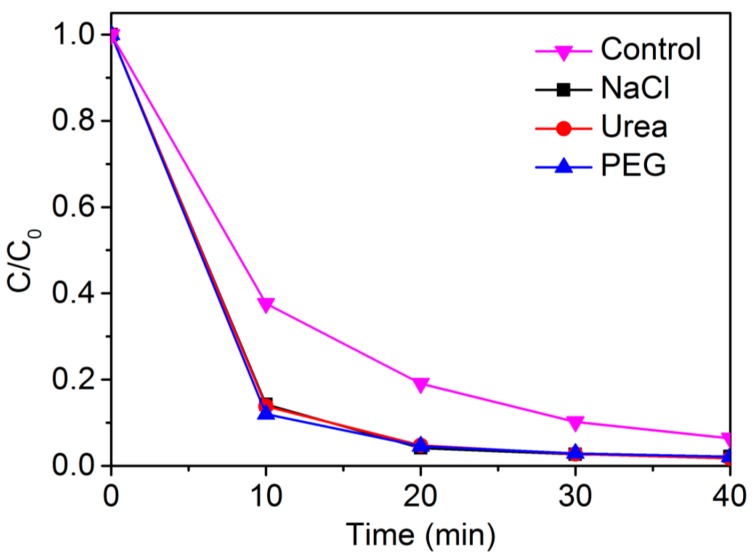
Concentration changes of AR1 in the presence of 0.10 mol/L NaCl, 0.10 mol/L Urea or 0.10 mol/L PEG at 25 °C with 4 × 10^−3^ mol/L H_2_O_2_ ([Co^III^(opba)]^−^-Py-MWCNTs = 0.2 g/L, pH 9.0 (0.01 mol/L borate buffer)).

It has been reported that Cl^−^ will bind to •OH radicals during the reaction process, causing a dramatic decrease in the detoxification rate [36,37]. NaCl, PEG-containing compounds and urea are necessary to industry manufacture. At the same time, PEG always presents as biodegradable surfactant or vulnerable constitutional unit in water systems. However, they cause severe difficulties for •OH-dominated reaction process in practical effluents [38,39]. To study the target micropollutant detoxification ability with [Co^III^(opba)]^−^-Py-MWCNTs in high backgrounds of complex constituents, we conducted detoxification experiments with the above-mentioned •OH-inhibiting industrial auxiliaries whose concentrations were two thousand-fold of the substrate (the molecular weight of PEG was 200). Extraordinarily, the three substances evidently improved the detoxification efficiency as shown in Figure 4.

To figure out this abnormal phenomenon, we put a sight into the physical adsorption between MWCNTs and AR1 in each impurity. The results showed that the adsorption of AR1 by MWCNTs was enhanced in the existence of NaCl, urea or PEG (Figure S9). According to reports, urea acting as a swelling agent for textile dyeing might have inflated the sectional area of MWCNTs, which improved the adsorption of AR1 [30]. As a surfactant, PEG reduced the interface state of solution and increased the dispersion of catalysts [40]. The acceleration in NaCl solution might be ascribed to the salting-out of substrate, which made AR1 more affine to solid catalyst. In conclusion, the bioinspired catalytic system transformed the negative effects of the external constituents that occurred in •OH-dominated reactions into positive factors for the oxidation of recalcitrant substrates.

### 2.4. Cyclic Catalytic Oxidation

The stability and cyclic catalytic oxidation are two fundamental requirements for heterogeneous catalyst. In our test, with sole presence of [Co^III^(opba)]^−^-Py-MWCNTs and AR1, an adsorption dynamic equilibrium happened in 20 min. Once H_2_O_2_ was added (Figure S10), a catalytic oxidation of substrate proceeded rapidly. Besides, the continuous cycle experiments were performed for eight times. For each run, the initial catalyst was regained by centrifugal separation and the same amount of AR1 and H_2_O_2_ was replenished to substrate solution. The reaction solution was also stirred for 60 min at 25 °C. As shown in Figure S11, the catalyst remained efficient after repetitive eight cycles, and the slight decay was largely ascribed to the mass loss of catalysts during centrifugal separation.

### 2.5. Mechanism and Pathway

#### 2.5.1. Mechanism

As an effective •OH scavenger, isopropanol (one thousand-fold of AR1) was introduced to elucidate the reactive species responsible for substrate detoxification [41,42]. As shown in Figure S12, both the two processes with or without isopropanol displayed no significant disparity. This result implied there was no free •OH involved in this system. To gain further insight into whether there formed short-lived free radicals during the catalytic oxidation in the [Co^III^(opba)]^−^-Py-MWCNTs/H_2_O_2_ system, EPR spin-trapping experiments with DMPO were conducted to provide useful information on the reaction mechanism, and the result is shown in Figure S13. As shown, nearly no DMPO-•OH signals were observed, confirming the existence of a non-hydroxyl radical process. Therefore, we speculated the reaction mechanism as high-valent cobalt-oxo oxidizing mechanism.

To study the function mechanism of the fifth ligand, different Py-based ligands were employed [43]. When electron-withdrawing 4-cyanopyridine (4-Cypy) was added into homogenous reaction system, prompt detoxification was achieved in 15 min, which performed totally opposite to the electron-donating 4-Ampy. The detoxification rate of AR1 for [Co^III^(opba)]^−^ with Py-based compound was in the order of 4-Cypy-[Co^III^(opba)]^−^ > [Co^III^(opba)]^−^ > Py-[Co^III^(opba)]^−^ > 4-Ampy-[Co^III^(opba)]^−^. The same discipline was similar to the detoxification of CIP (Figures S14 and S15). It turned out that the employment of fifth ligand was a promising approach to control the progress of metal complex’s oxidizing reactions.

From the point of characteristic electronic structures, the critical role of the fifth ligand in their functionalities of organic metal complex catalysis might be clearly illuminated [44]. In this paper, DFT calculations and frontier molecular orbitals theory were employed to investigate. Considering that less than five spin electrons exist in Co d orbitals, all DFT calculations using Gaussian 09 program package and 6–31 G basis set were performed at the UB3LYP level with spin states of S = 0, S = 1 or S = 2 [45]. Their total energy is shown in Table S1, and we chose models with S = 1 due to their lowest total energy which implied that the spin state with S = 1 was the most reasonable existence for this cobalt complex. The optimized structure analysis (Figure S16) revealed that the distance between Co and O was about 1.68 Å, conforming to the length of double bond (Table S2). Furthermore, almost half of their electron spin density distribution (~1.1) located at the O site, which means a single spin electron located around O atom. So, in this paper, we portrayed the active species as Co^IV^=O•. Furthermore, the resulting Co=O bond length of 4-Cypy-[•O=Co^IV^(opba)]^−^ was 1.67904 Å, shorter than that of Py-[•O=Co^IV^(opba)]^−^ (1.68345 Å), and the longest was that of 4-Ampy- [•O=Co^IV^(opba)]^−^ (1.68693 Å) (see structure in Tables S3–S6). Notably, the Co=O bond length of [•O=Co^IV^(opba)]^−^-Py-MWCNTs was longer than that of 4-Cypy-[•O=Co^IV^(opba)]^−^ but shorter than that of Py-[•O=Co^IV^(opba)]^−^. This intriguing consequence ascribed to the contribution of MWCNTs, which means the introduction of MWCNTs weakened the electron-donating ability of Py and achieved efficient catalytic oxidation of substrates. The electron spin density distribution order of 4-Cypy-[•O=Co^IV^(opba)]^−^ < [•O=Co^IV^(opba)]^−^-Py-MWCNTs < Py-[•O=Co^IV^(opba)]^−^ < 4-Ampy-[•O=Co^IV^(opba)]^−^ (Tables S7–S10) announced that the efficiency disparity of [Co^III^(opba)]^−^ with different ligands was ascribed to the electronegativity effect of the fifth ligands on the Co=O moiety, thus distinguishable hydrogen peroxide activation ability and efficiency occurred. Meanwhile, the spin populations were predominantly located around the Co=O moiety, achieving an electrophilic attack on the electron-rich chemical bond and the aromatic ring of substrates [30].

**Figure 5:**
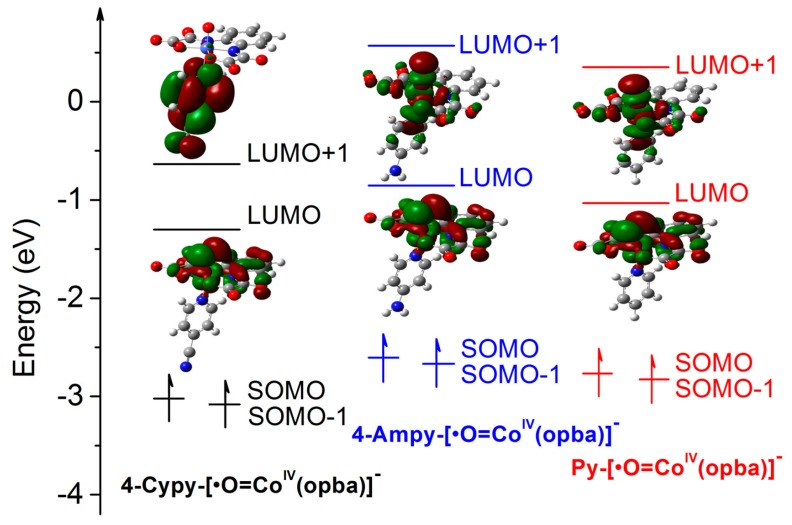
Energy level scheme for 4-Cypy-[•O=Co^IV^(opba)]^−^, 4-Ampy-[•O=Co^IV^(opba)]^−^ and Py-[•O=Co^IV^(opba)]^−^ (The inset electronic structures were the levels around the LUMO and LUMO+1).

Furthermore, the electronic structure around the singly occupied molecular orbital (SOMO) and the lowest unoccupied molecular orbital (LUMO) had been calculated (Figure 5). The resulting levels around the SOMO and LUMO were in the order of 4-Cypy-[•O=Co^IV^(opba)]^−^ < Py-[•O=Co^IV^(opba)]^−^ < 4-Ampy-[•O=Co^IV^(opba)]^−^. With the lowest energy level, 4-Cypy- [•O=Co^IV^(opba)]^−^ was most vulnerable to substrates, thus presented a higher detoxification efficiency. It was inferred that the electron-deficient property of the fifth ligand was beneficial for inward pulling the excess electron, which decreased the stability of the cobalt plane, resulting to the lowered energy level SOMO and LUMO of active intermediates [46]. However, an intriguing electronic structure change around the LUMO+1 triggered by 4-Cypy appeared, which means the electron deficient 4-Cypy was chemically active and may be oxidized by the active 4-Cypy-[•O=Co^IV^(opba)]^−^. This conclusion was confirmed by the concentration decrease of 4-Cypy in the reacting solution during the detoxification of CIP (Figure S17). When the coordinated 4-Cypy was consumed, free 4-Cypy in reacting solution would take the place of the old, and finally resulted in the decreasing concentration of 4-Cypy in solution. This was the reason why we employed Py but not Cypy as the fifth ligand for catalyst support. To the best of our knowledge, no researchers have reported the unique LUMO+1 orbital and reaction characteristics 4-Cypy in metal-oxo catalytic systems, and this might be a meaningful discovery needing further investigation.

**Figure 6:**
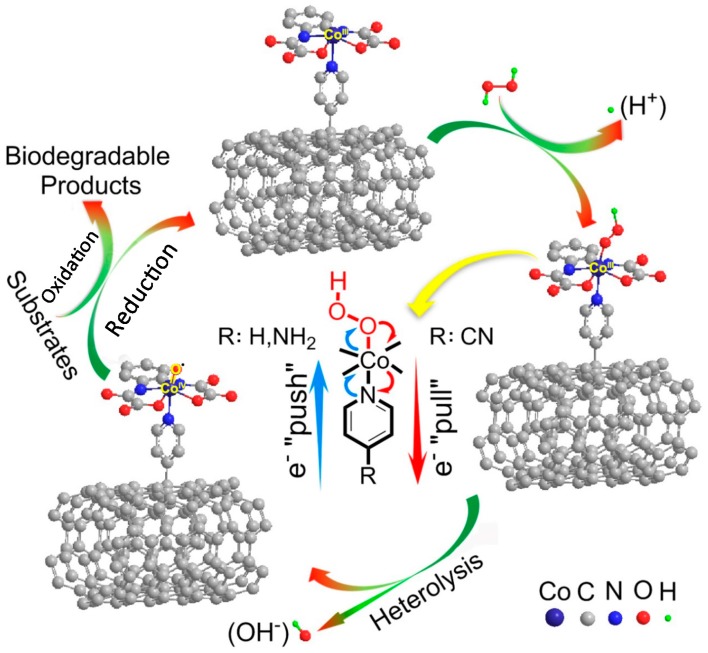
Proposed reaction mechanism of substrates over [Co^III^(opba)]^−^-Py-MWCNTs.

According to the radical trapping tests, EPR results and DFT calculations, non-hydroxyl radical catalytic processes could be proved, and high-valent cobalt-oxo species were speculated. As illustrated in Figure 6, in alkaline pH, the nucleophilic HOO– of the ionized H_2_O_2_ was coordinated with the center Co by vacant orbital, followed by the heterolytical cleavage of O–O bond and forming high-valent cobalt-oxo active species. When [Co^III^(opba)]^−^ was coordinated with electron-withdrawing or -donating ligands, the electron density population of Co was changed, and the cleavage of O–O bond was influenced self-evidently. If combined with electron-withdrawing ligand such as 4-Cypy, [Co^IV^=O•]^−^ possessed an enhanced electrophilicity and stronger oxidizing power with deficient electron density on Co=O moiety [47].

#### 2.5.2. Detoxification Pathway

**Figure 7:**
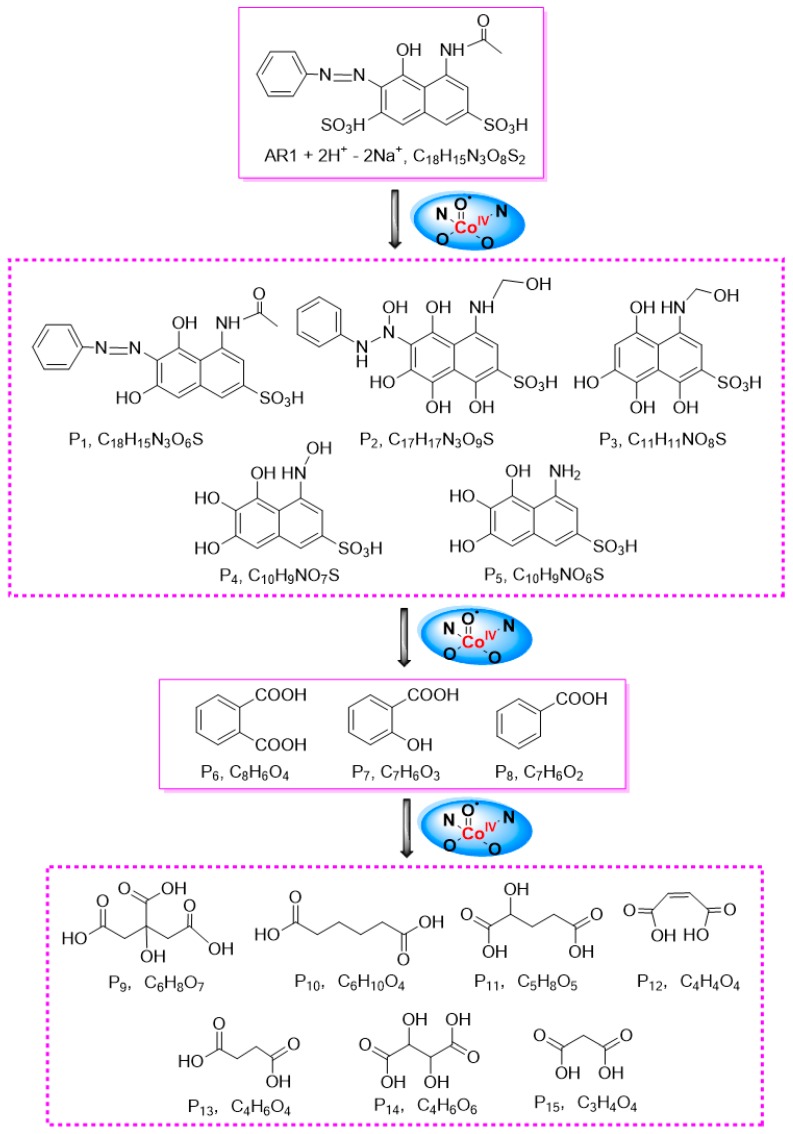
Possible detoxification pathway for the catalytic oxidation of AR1.

To identify the detoxification pathway of substrate, the final solution in the catalytic system with [Co^III^(opba)]^−^-Py- MWCNTs was concentrated by vacuum freeze-drying and examined by LC–MS in negative ion mode. The detected intermediates and final products are shown in Figure 7 and Table S11. According to reports, [Co^IV^=O•]^−^ was responsible for inserting an oxygen into chemical bonds of the substrates. Theoretically, when catalyst encountered with substrate, electron-rich chemical bond of substrate should be the first to be inserted with an O atom, followed by formations of OH-containing compounds [48,49]. From the detected OH-containing products, we speculated that the oxygen atom transfer reaction occurred due to high-valent cobalt-oxo active species, and produced naphthalene ring-containing compounds (P1–P5) at first. Then the resulting products were oxidized to benzoic acid derivatives (P6–P8), and finally oxidized to some kinds of low molecular-weight organic acids (P9–P15) because of aromatic ring-opening reaction. All these final small molecule acids (P9–P15) were non-poisonous and could be used to feed microorganisms [30].

## 3. Materials and Methods

### 3.1. Synthesis of Catalyst

4-aminopyridine (4-Ampy) was grafted onto MWCTNs through diazonium salt method [50]: 10 g 4-Ampy was dissolved in 4 mol/L hydrochloride solution (30 mL), and the resulting mixture was kept in an ice water bath. 8 mol/L NaNO_2_ aqueous solution (7 mL) was dropwise added into 4-aminopyridine solution with vigorous stirring at 0 °C for 30 min. 50 mg MWCNTs was quickly homogenized in 200 mL DMF by ultrasonic dispersing, and the temperature was decreased to 0 °C under an ice water bath. Finally, 4-Ampy/NaNO_2_ mixture was added into the DMF suspension by drops. After stirring for 3 h under ice water bath, then heating up to room temperature for another 15 h, the Py-modified multiwalled nanotubes (Py-MWCNTs) were filtered out with high-speed freezing centrifuge, and washed by HCl, NaOH solution and deionized water, acetone respectively. Then the drying Py-MWCNTs (50 mg) were added into a three-mouth flask, and ultrasonic dispersed for 2 h in 120 mL methanol. This was followed by adding 20 mg Na[Co^III^(opba)] and refluxing for 2 h. Then the Py-modified multiwalled carbon nanotubes-supported cobalt(III) oxamate complex ([Co^III^(opba)]^−^-Py-MWCNTs) was obtained and filtered out by centrifuging and washed by methanol and acetone until the solution turned to colorless, then it was separated and dried in vacuum. Scheme S1 illustrates the reaction processes.

### 3.2. Catalytic Experiments and Analysis

The typical experimental procedure for the catalytic oxidation of AR1 and CIP was conducted with [Co^III^(opba)]^−^-Py-MWCNTs and carried out in a 40 mL test tube. The reaction temperature was maintained at 25 °C (AR1) or 45 °C (CIP) by means of a constant temperature water bath. The initial concentration of AR1 or CIP in 0.01 M borate buffer was 50 μM with 0.2 g/L [Co^III^(opba)]^−^-Py-MWCNTs, and then a certain amount of H_2_O_2_ was added to the reaction solution. At given time intervals, a small amount of reaction mixture of AR1 was frequently taken out for observing the concentration changes of substrates, and the concentration of dyes was determined using an ultraviolet-visible (UV-vis) spectrometer (Hitachi U-3010, Tokyo, Japan). The concentration changes of CIP were analyzed by ultra-performance liquid chromatography (UPLC, Acquity BEH C18 column (1.7 μm, 2.1 × 50 mm), Waters, Milford, MA, USA). The removal rate was calculated by Equation (1), where C is the concentration of AR1 or CIP at each reaction time and C_0_ is the initial concentration of the substrate.

Residual rate = C/C_0_ (1)

### 3.3. Characterization

X-ray photoelectron spectroscopy (XPS, Thermo Fisher Scientific, Waltham, MA, USA) data were recorded with the Thermo Scientific K-Alpha spectrometer (monochromatic Al Kα, 1486.6 eV) and analyzed with Avantage (Thermo Fisher Scientific, Waltham, MA, USA), and the binding energy peaks of the XPS spectra were calibrated by placing the principal C1s binding energy peak at 284.7 eV. For ultraviolet spectroscopy (UV-vis, U-3010, Hitachi Corporation, Tokyo, Japan) detection, MWCNTs, Py-MWCNTs, Coopba, Et_2_H_2_opba and [Co^III^(opba)]^−^-Py-MWCNTs was dispersed in acetonitrile and water (V(CH_3_CN):V(H_2_O) = 1:100). Thermogravimetric analysis (TGA, Mettler Toledo, Zurich, Switzerland) detection was carried out at a heating rate of 5 °C/min in 40 mL/min nitrogen flow. Microwave digestion-flame atomic absorption spectrometry (AAS, Sollar M6, Thermo Fisher Scientific, Waltham, MA, USA) was employed for the measurement of Co content.

UV-vis Spectrum and ultra-performance liquid chromatography (UPLC, Acquity BEH C18 column (1.7 μm, 2.1 × 100 mm), Waters, Milford, MA, USA) were employed for the immediate analyses. Detection was accomplished using a photodiode array detector (PDA, Waters, Milford, MA, USA) set at 278.6 nm for CIP, 371 nm for AR1 and 279.2 nm for 4-cyanopyridine. The binary system phases were 13% (A) acetonitrile and 87% (B) formic acid and water (1:1000, *V/V*) with a flow rate of 0.25 mL/min for CIP, while for AR1 the ratio was 19% A (acetonitrile) and 81% B (50 × 10^−3^ mol/L ammonium acetate and 2 × 10^−3^ mol/L tetrabutylammonium bromide) at 0.25 mL/min. We detected the oxidative intermediates of AR1 using LC–MS (UPLC/Synapt G2-S HDMS, Waters, Milford, MA, USA) after reacted for 60 min.

A Bruker A300 spectrometer (Bruker, Karlsruhe, Baden-Württemberg, Germany) was used to record the electron paramagnetic resonance (EPR) signals of 5,5-dimethyl-pyrroline-oxide (DMPO) at 20 °C with the following settings: center field, 3508 G; sweep width, 80 G; microwave frequency, 9.88 GHz; modulation frequency, 100 kHz; power, 20 mW. The active intermediates were prepared from 2 × 10^−5^ mol/L solution of complex 1 in acetonitrile with slight ultrapure water at −40 °C (the pH value was adjusted to 9.5 with ammonia water previously) using 2 equivalents of 3-chloroperoxybenzoic acid (mCPBA, 85%), and the solution was infused directly into ion source of high-definition mass spectrometry with a syringe manually. In addition, to determine active intermediates, the source temperature was set at 40 °C to minimize the decomposition of intermediate species.

## 4. Conclusions

In summary, we constructed a bioenzyme mimic using MWCNTs grafted oxamate cobalt(III) complex to eliminate target substrate in the presence of H_2_O_2_. In [Co^III^(opba)]^−^-Py- MWCNTs/H_2_O_2_ system, pyridine-based compound acted as the fifth ligand, and MWCNTs played as the protein structure. This system presented a good oxidation activity in micropollutant removal under mild conditions with good recyclability. And its activity was obviously improved in high backgrounds of complex constituents because of the enhanced capture ability of MWCNTs. The oxygen atom transfer products were all bio-degradable small molecule acids, which kept pace with the concept of green chemistry. DFT calculations were employed to explain the reacting mechanisms, and they showed that electron-deficient ligand and support weakened the electron density on Co=O segment of oxamate cobalt(III) intermediate which enhanced the oxidizing power of the active center. This paper suggested that controllable oxidant activation may be available through introducing appropriate ligand and support with certain electron nature to metal complex, and it might offer an arena for the chemist to develop needed catalyst.

## Supplementary Materials

The following are available online at www.mdpi.com/1996-1944/10/10/1169/s1. Carbon-based Oxamate Cobalt(III) Complexes as Artificial Enzyme for Contaminant Elimination in High Backgrounds of Complicated Constituents. Figure S1: Thermogravimetric analysis of pristine MWCNTs, Py-MWCNTs, [Co^III^(opba)]^−^-Py- MWCNTs and [Co^III^(opba)]^−^. Atmosphere: N_2_ gas, Rate: 5 °C/min. Figure S2: The standard working curve of Cobalt ions in atomic absorption spectrometry. Figure S3: Curve fitting of Co2p peaks of MWCNTs, Py-MWCNT, [Co^III^(opba)]^−^ and [Co^III^(opba)]^−^-Py-MWCNTs. Figure S4: Curve fitting of C1s peaks of MWCNTs, Py-MWCNT, [Co^III^(opba)]^−^ and [Co^III^(opba)]^−^-Py-MWCNTs. Figure S5: The effect of catalyst system pH on AR1 removal at 25 °C with 4.0 × 10^−3^ mol/L H_2_O_2_, [Co^III^(opba)]^−^-Py-MWCNTs = 0.2 g/L). Figure S6: Effect of concentration of H_2_O_2_ on detoxification of AR1 ([Co^III^(opba)]^−^-Py-MWCNTs = 0.2 g/L, T = 25 °C, pH 9.0 (0.01 M borate buffer)). Figure S7: Effect of temperature on detoxification of AR1 with 4.0 × 10^−3^ mol/L H_2_O_2_ ([Co^III^(opba)]^−^-Py-MWCNTs = 0.2 g/L, pH 9.0 (0.01 M borate buffer)). Figure S8. The concentration changes of CIP (5.0 × 10^−5^ mol/L) with 1.0 × 10^−2^ mol/L H_2_O_2_, pH 8.2 (0.01 M borate buffer). Figure S9: The concentration changes of AR1 (5.0 × 10^−5^ mol/L) with 0.19 g/L MWCNTs (the same MWCNTs amount as [Co^III^(opba)]^−^-Py-MWCNTs) at 25 °C, pH 9.0 (0.01 M borate buffer). Figure S10: Concentration changes of AR1 after adsorption equilibrium with or without 4.0 × 10^−3^ mol/L H_2_O_2_ ([Co^III^(opba)]^−^-Py-MWCNTs = 0.2 g/L, T = 25 °C, pH 9.0 (0.01 M borate buffer)). Figure S11: The cyclic catalytic oxidation of AR1 with 4.0 × 10^−3^ mol/L H_2_O_2_ after 60 min ([Co^III^(opba)]^−^-Py-MWCNTs = 0.2 g/L, T = 25 °C, pH 9.0 (0.01 mol/L borate buffer)). Figure S12: Concentration changes of AR1 in the presence of [Co^III^(opba)]^−^-Py-MWCNTs with isopropanol or without isopropanol with 4.0 × 10^−3^ mol/L H_2_O_2_ ([Co^III^(opba)]^−^-Py-MWCNTs = 0.2 g/L, T = 25 °C, pH 9.0 (0.01 mol/L borate buffer)). Figure S13: DMPO spin-trapping EPR spectra in the presence of [Co^III^(opba)]^−^-Py-MWCNTs (0.2 g/L), [H_2_O_2_] = 1.0 × 10^−2^ mol/L, [DMPO] = 5.0 × 10^−3^ mol/L. Experimental data recorded at 20 °C. Figure S14: Concentration changes of CIP with 5.45 × 10^−3^ mol/L pyridine or 4-cyanopyridine and 1.0 × 10^−2^ mol/L H_2_O_2_ at 45 °C ([Co^III^(opba)]^−^-Py-MWCNTs = 0.2 g/L, pH 8.2 (0.01 M borate buffer)). Figure S15: Oxidation of AR1 with different fifth ligands (50 folds as the concentration of [Co^III^(opba)]^−^) in 5 min (a) and 30 min (b) with 4.0 × 10^−3^ mol/L H_2_O_2_ ([Co^III^(opba)]^−^ = 1.09 × 10^−4^ mol/L, T = 25 °C, pH 9.0 (0.01 M borate buffer)). Figure S16: Electron spin population of [•O=Co^IV^(opba)]^−^-Py-MWCNTs (a), 4-Cypy-[•O=Co^IV^(opba)]^−^ (b), Py-[•O=Co^IV^(opba)]^−^ (c) and 4-Ampy- [•O=Co^IV^(opba)]^−^ (d) calculated by DFT structures (S = 1). Figure S17: Concentration changes of 4-cyanopyridine in detoxification of CIP with 1.0 × 10^−2^ mol/L H_2_O_2_ at 45 °C ([Co^III^(opba)]^−^-Py-MWCNTs = 0.2 g/L, pH 8.2 (0.01 M borate buffer)). Table S1: The total electron energy (a.u.) of 4-Cypy-[•O=Co^IV^(opba)]^−^, Py-[•O=Co^IV^(opba)]^−^, 4-Ampy- [•O=Co^IV^(opba)]^−^ and [•O=Co^IV^(opba)]^−^_Py_MWCNTs. Table S2: The Co–O bond length of 4-Cypy-[•O=Co^IV^(opba)]^−^, Py-[•O=Co^IV^(opba)]^−^, 4-Ampy-[•O=Co^IV^(opba)]^−^ and MWCNTs-Py- [•O=Co^IV^(opba)]^−^ calculated by DFT (S = 1). Table S3: The optimized structure of MWCNTs-Py- [•O=Co^IV^(opba)]^−^ calculated by DFT (S = 1).Table S4: The optimized structure of 4-Cypy-[•O=Co^IV^(opba)]^−^ calculated by DFT (S = 1). Table S5: The optimized structure of 4-Ampy-[•O=Co^IV^(opba)]^−^ calculated by DFT (S = 1). Table S6: The optimized structure of Py-[•O=Co^IV^(opba)]^−^ calculated by DFT (S = 1). Table S7: Mulliken spin densities of [•O=Co^IV^(opba)]^−^-Py-MWCNTs (S = 1). Table S8: Mulliken spin densities of 4-Cypy-[•O=Co^IV^(opba)]^−^ (S = 1). Table S9: Mulliken spin densities of Py-[•O=Co^IV^(opba)]^−^ (S = 1). Table S10: Mulliken spin densities of 4-Ampy-[•O=Co^IV^(opba)]^−^ (S = 1). Table S11: Oxidative intermediates of AR1 in the presence of [Co^III^(opba)]^−^-Py-MWCNTs and H_2_O_2_ examined by UPLC Synapt G2-S HDMS in the negative ion mode after 60 min of reaction time. Scheme S1. The preparation of [Co^III^(opba)]^−^-Py-MWCNTs.
