# Supplementary material for: Carbon-Based Oxamate Cobalt(III) Complexes as Bioenzyme Mimics for Contaminant Elimination in High Backgrounds of Complicated Constituents
Source: Materials (Basel). 2017 Oct 12;10(10):1169. doi: 10.3390/ma10101169 (PMC5666975; doi:10.3390/ma10101169)
Supplement: Supplementary file 1 [file materials-10-01169-s001.pdf]

materials

Supplementary Materials

Carbon-based Oxamate Cobalt(III) Complexes as bioenzyme mimics for Contaminant Elimination in High Backgrounds of Complicated Constituents

Nan Li [1,*], Yun Zheng [1], Xuemei Jiang [1], Ran Zhang [1], Kemei Pei [2] and Wenxing Chen [1,*]

[1] National Engineering Lab for Textile Fiber Materials & Processing Technology (Zhejiang), Zhejiang Sci-Tech University, Hangzhou 310018, China;
[2] Department of Chemistry, Zhejiang Sci-Tech University, Hangzhou 310018, China
* Correspondence: linan@zstu.edu.cn (N.L.); wxchen@zstu.edu.cn (W.C.);
Tel.: +86-571-8684-3611 (W.C.); Fax: +86-571-8684-3611 (W.C.)

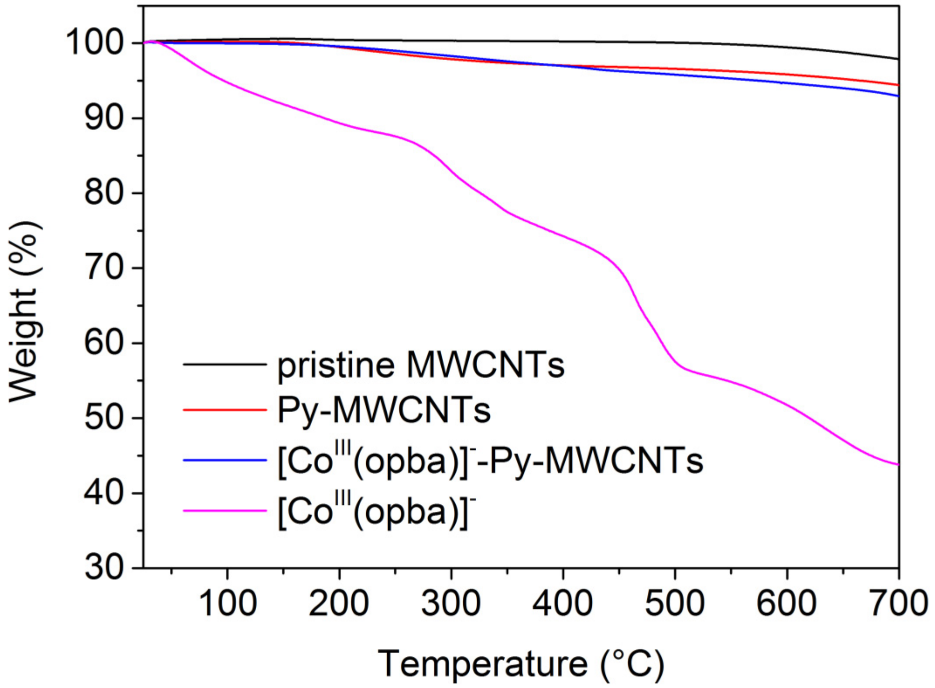

Figure S1. Thermogravimetric analysis of pristine MWCNTs, Py-MWCNTs, $[Co^{III}(opba)]^-$-Py-MWCNTs and $[Co^{III}(opba)]^-$. Atmosphere: N_2 gas, Rate: 5 °C/min.

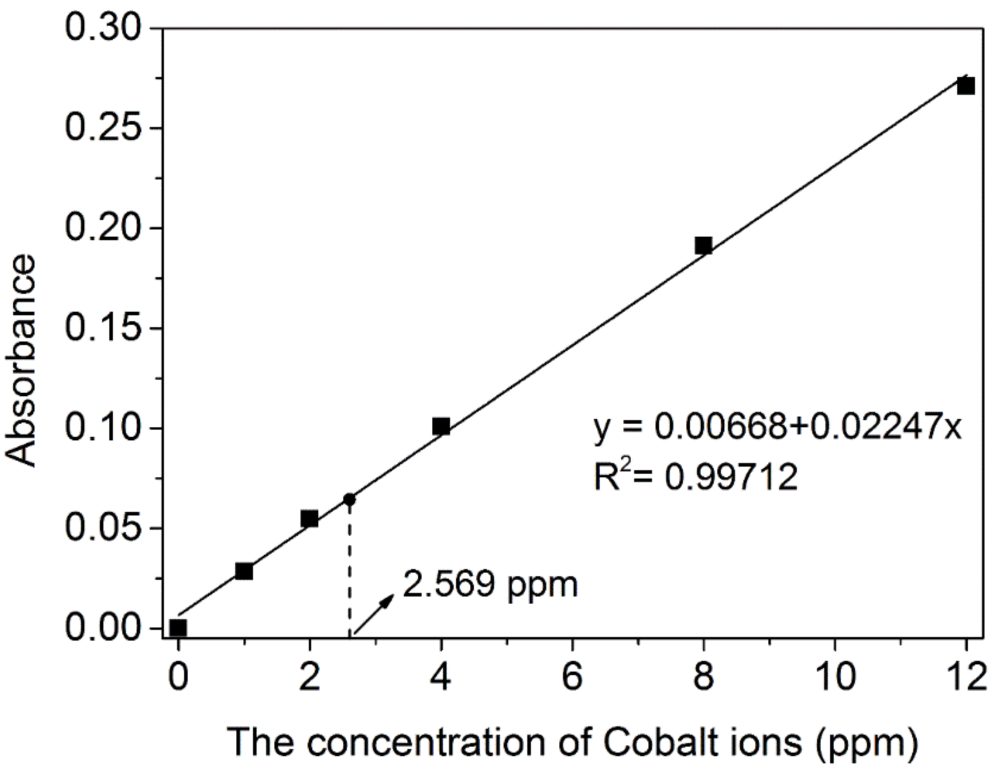

Figure S2. The standard working curve of Cobalt ions in atomic absorption spectrometry.

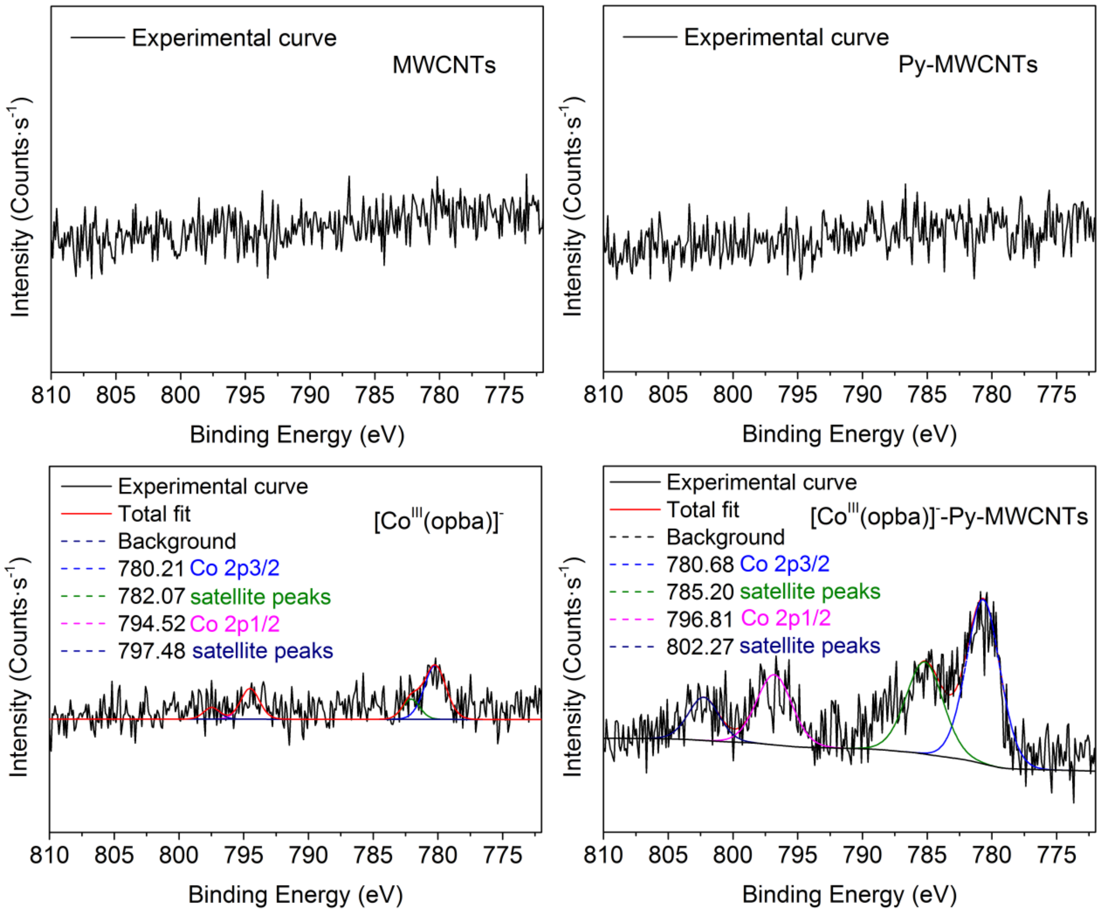

Figure S3. Curve fitting of Co2p peaks of MWCNTs, Py-MWCNT, $[Co^{III}(opba)]^-$ and $[Co^{III}(opba)]^-$-Py-MWCNTs.

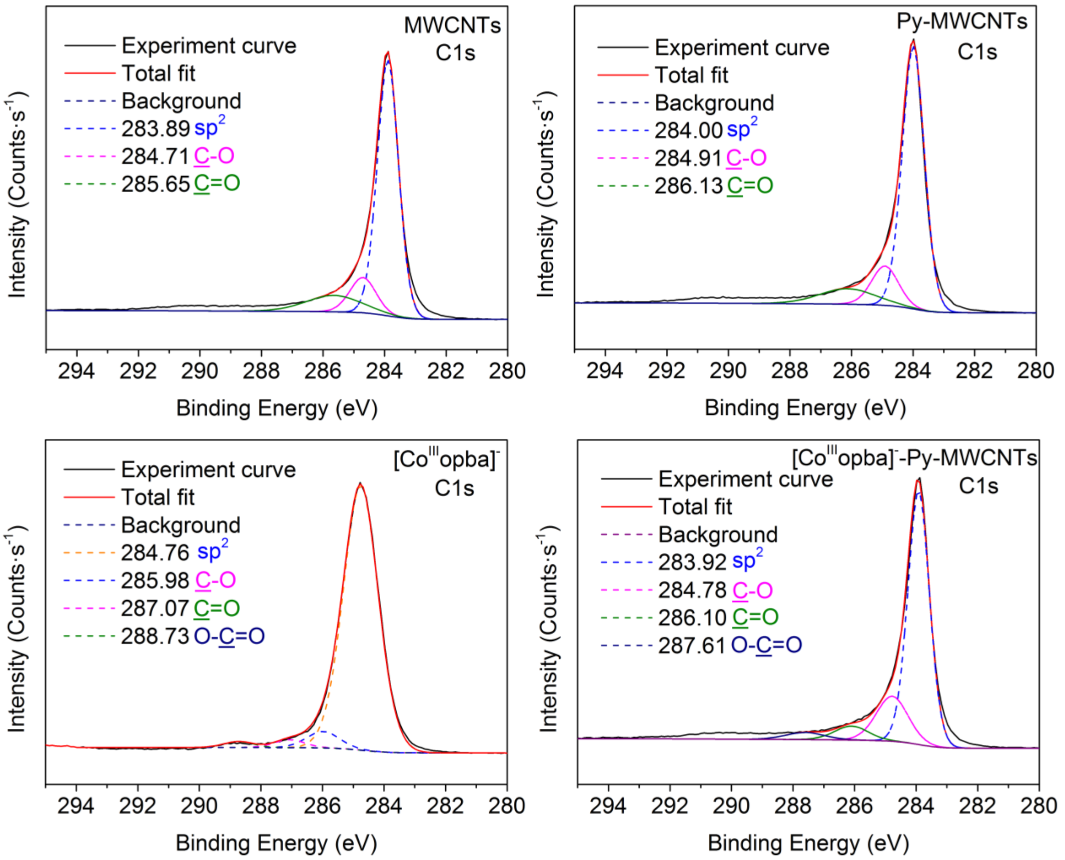

Figure S4. Curve fitting of C1s peaks of MWCNTs, Py-MWCNT, $[Co^{III}(opba)]^{-}$ and $[Co^{III}(opba)]^{-}$-Py-MWCNTs.

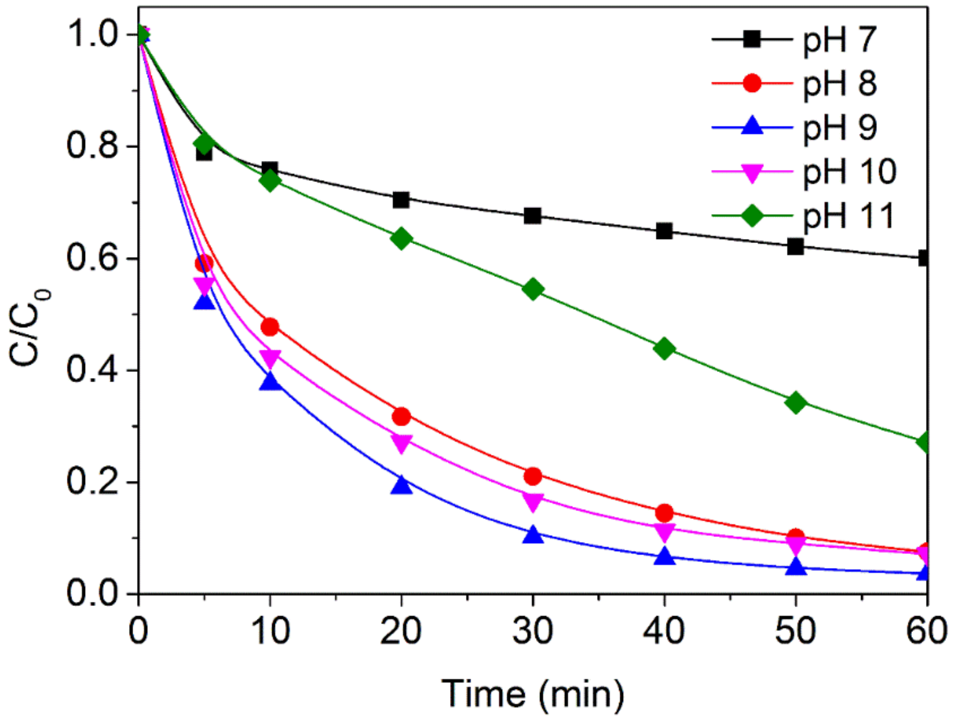

Figure S5. The effect of catalyst system pH on AR1 removal at 25 °C with 4.0×10^{-3} mol/L H_2O_2, ($[Co^{III}(opba)]^{-}$-Py-MWCNTs = 0.2 g/L).

Natural water is slightly alkaline, hence the effect of solution pH on the rate of substrate decomposition was critical [1]. Here we conducted a set of experiments where the solution pH was adjusted to 7-11 using H_2SO_4 (0.10 mol/L) and NaOH (0.10 mol/L) in 0.01 mol/L borate buffer. The results of the conversion rates are depicted in **Figure S5**. We can see that $[Co^{III}(opba)]^-$-Py-MWCNTs/H_2O_2 catalytic system could proceed at a pH range from 8 to 11 and the maximum rate occurred at pH 9.0-9.5 which was applicable to practical waste water purification [2]. Such catalytic process was different from the traditional hydroxyl radical process where the oxidation can only take place under acidic pH condition. Meanwhile the inefficiency at pH 7 and extreme alkalinity could be interpreted as follows: H_2O_2 was more difficult to ionize when pH values were close to neutral, which limited the coordination between HOO^- and the central cobalt ions, thus inhibiting the formation of active species [3-5]. However, when the solution is strongly alkaline, HOO^-, the function anion, reacted with a H_2O_2 molecular and ultimately leaded to the generation of oxygen and water [6].

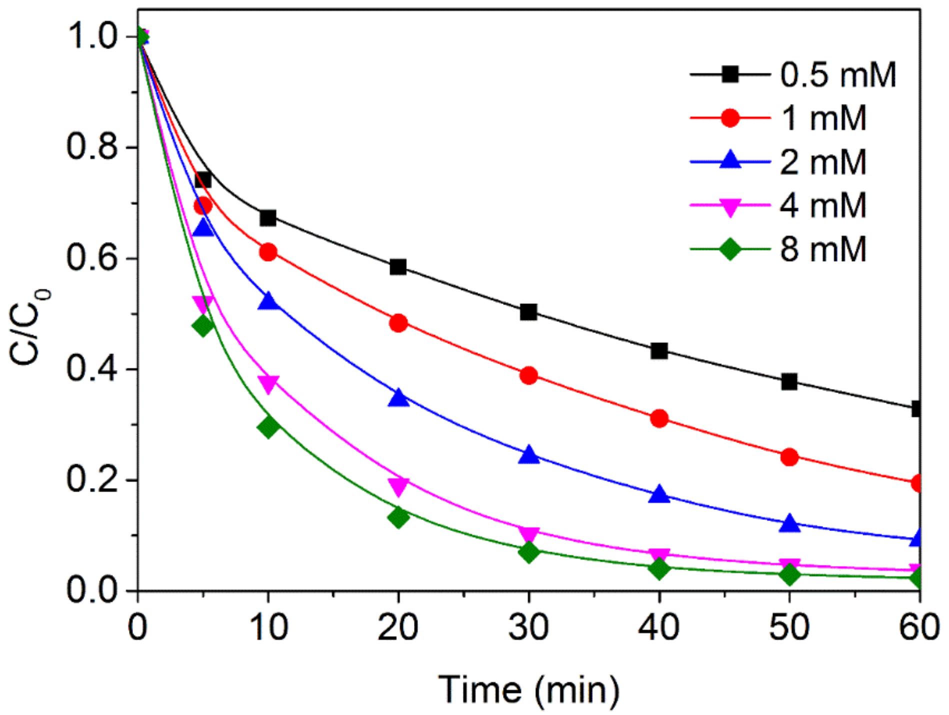

Figure S6. Effect of concentration of H_2O_2 on degradation of AR1 ($[Co^{III}(opba)]^-$-Py-MWCNTs = 0.2 g/L, T = 25 °C, pH 9.0 (0.01 M borate buffer)).

Besides, the degradation rate could be accelerated by the increase of H_2O_2 concentration. However, when the initial amount of H_2O_2 was added to 8.0×10^{-3} mol/L, the reaction rate did not significantly increase compared with the original concentration of 4.0×10^{-3} mol/L (see support information in **Figure S6**). At low H_2O_2 concentration, the number of generated active species was insufficient, and it increased with the extra addition of H_2O_2 until it reached a saturation point.

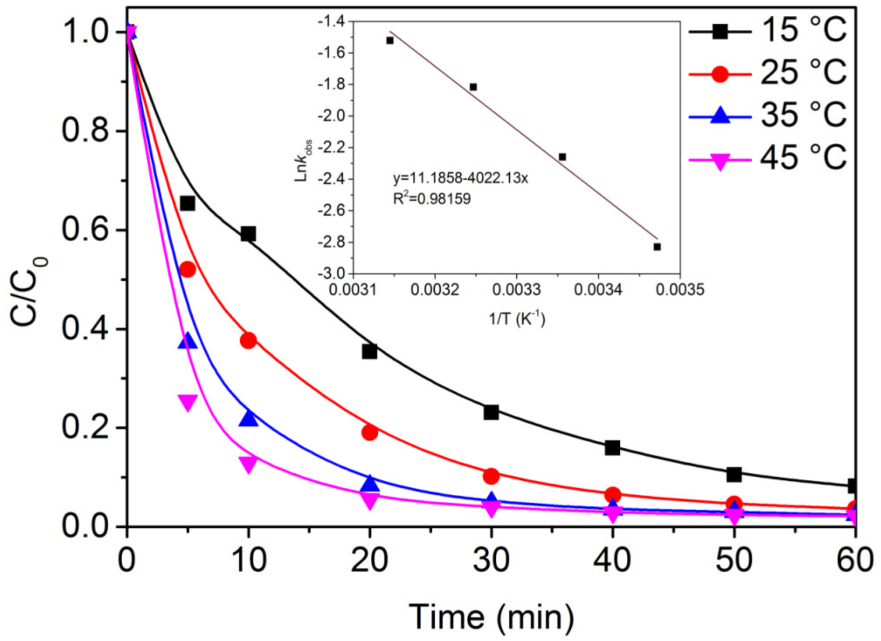

Figure S7. Effect of temperature on degradation of AR1 with 4.0×10^{-3} mol/L H_2O_2 ([Co^{III}(opba)]$^-$-Py-MWCNTs = 0.2 g/L, pH 9.0 (0.01 M borate buffer)).

Experiments in a temperature range from 15 °C to 45 °C were carried out as well to testify the applicability. Theoretically, a higher temperature would increase the collision rate between oxidants, catalyst and substrates, resulting in a higher catalytic velocity [7]. As expected, high temperature significantly speeded up the initial reaction rate. In the incipient reacting period, H_2O_2 was adequate and temperature was the decisive factor to substrate oxidation. And the initial apparent activation energy was utilized for the kinetic study. According to time courses of ln(C_0/C) at different temperatures, an Arrhenius plot (lnk_{obs}. 1/T) is presented in **Figure S7**, in which the slope of the straight line is proportional to the activation energy (E_a). The E_a of the catalytic process of AR1 in [Co^{III}(opba)]$^-$-Py-MWCNTs/H_2O_2 system was 33.40 KJ/mol, which announced the feasibility for the treatment of wastewater at relatively low temperature in comparison with ordinary oxidation reactions [8].

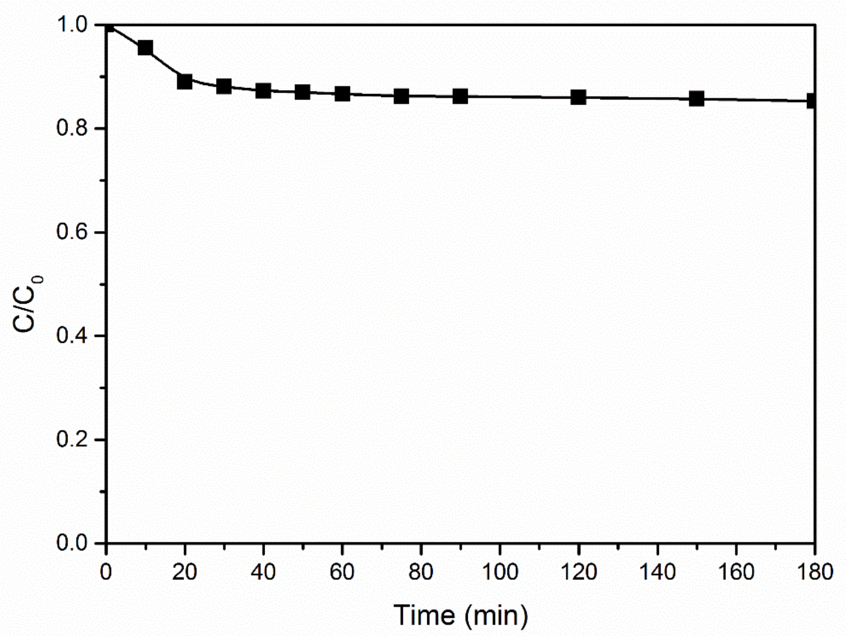

Figure S8. The concentration changes of CIP (5.0×10^{-5} mol/L) with 1.0×10^{-2} mol/L H_2O_2 at 45 °C, pH 8.2 (0.01 M borate buffer)

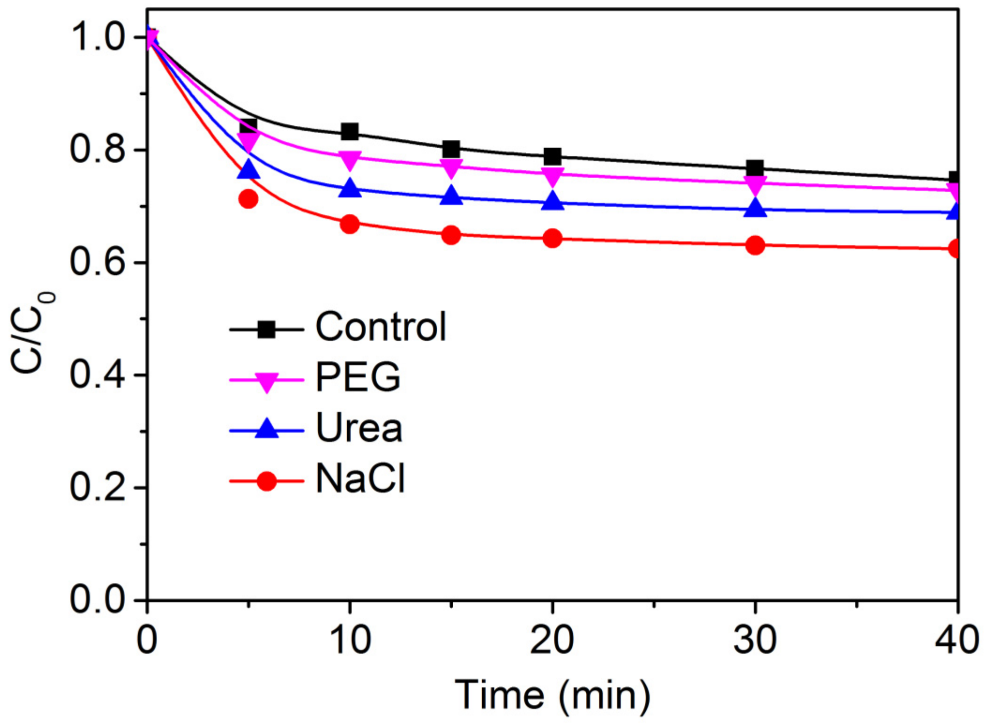

Figure S9. The concentration changes of AR1 (5.0×10^{-5} mol/L) with 0.19 g/L MWCNTs (the same MWCNTs amount as $[Co^{III}(opba)]^{-}$-Py-MWCNTs) at 25 °C, pH 9.0 (0.01 M borate buffer).

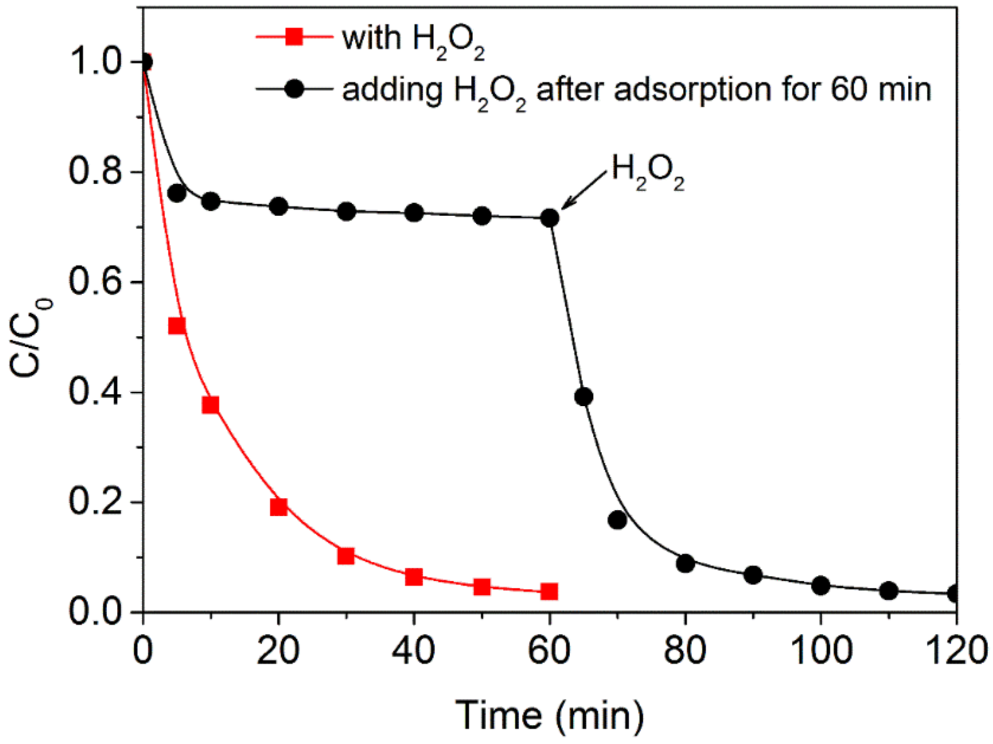

Figure S10. Concentration changes of AR1 after adsorption equilibrium with or without 4.0×10^{-3} mol/L H_2O_2 ($[Co^{III}(opba)]^{-}$-Py-MWCNTs = 0.2 g/L, T = 25 °C, pH 9.0 (0.01 M borate buffer)).

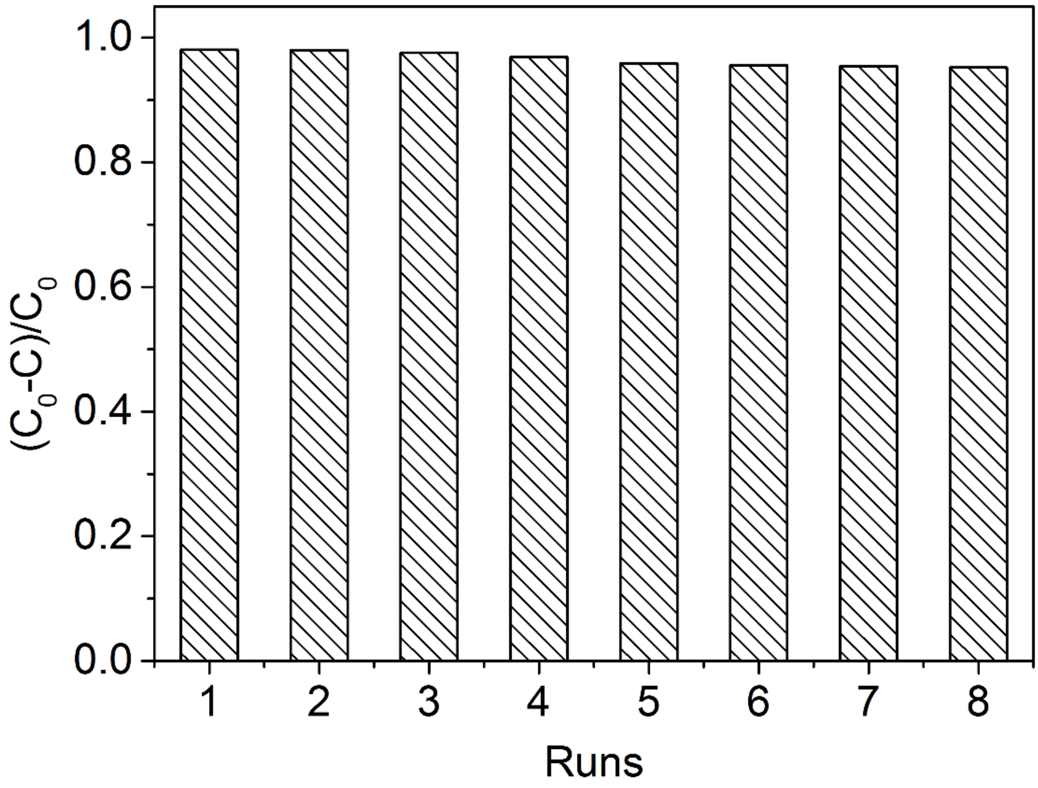

Figure S11. The cyclic catalytic oxidation of AR1 with 4.0×10⁻³ mol/L H_2O_2 after 60 min (the initial concentration of [Co^{III}(opba)]⁻-Py-MWCNTs = 0.2 g/L, T = 25 °C, pH 9.0 (0.01 mol/L borate buffer)).

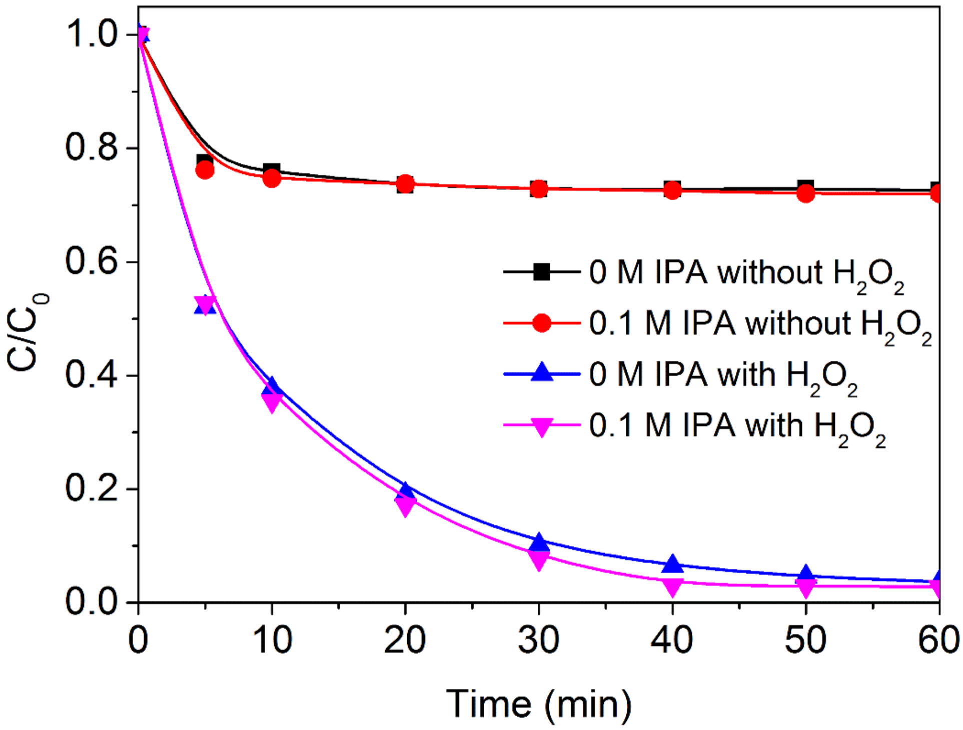

Figure S12. Concentration changes of AR1 in the presence of [Co^{III}(opba)]⁻-Py-MWCNTs with isopropanol or without isopropanol with 4.0×10^{-3} mol/L H_2O_2 ([Co^{III}(opba)]⁻-Py-MWCNTs = 0.2 g/L, T = 25 °C, pH 9.0 (0.01 mol/L borate buffer)).

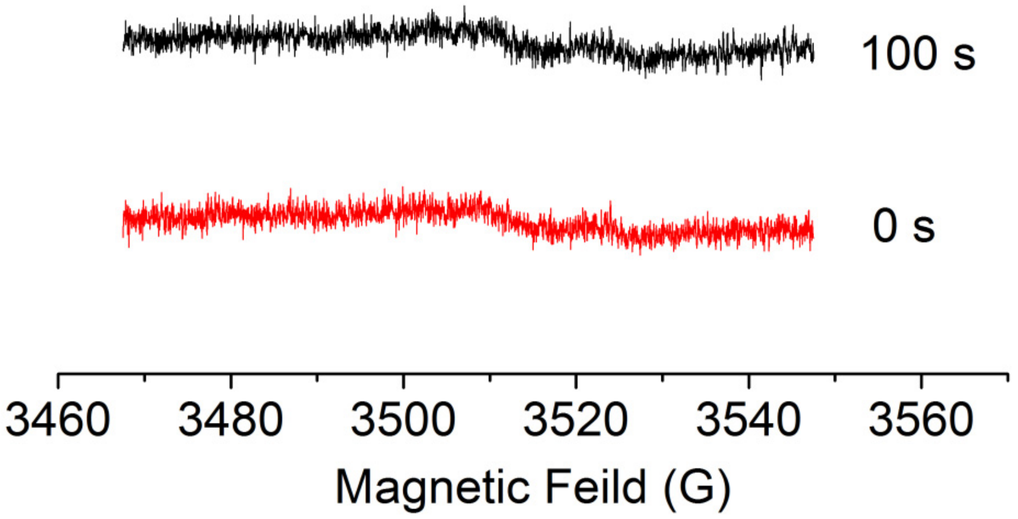

Figure S13. DMPO spin-trapping EPR spectra in the presence of $[Co^{III}(opba)]^{-}$-Py-MWCNTs (0.2 g/L), $[H_2O_2] = 1.0\times10^{-2}$ mol/L, $[DMPO] = 5.0\times10^{-3}$ mol/L. Experimental data recorded at 20 °C.

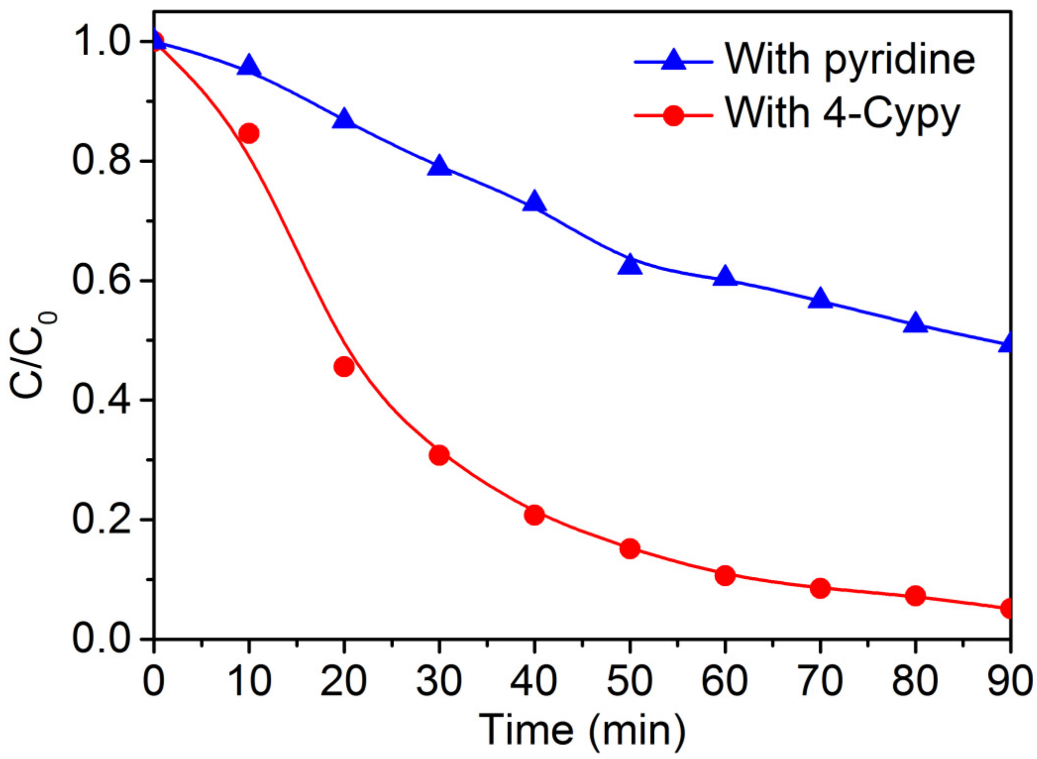

Figure S14. Concentration changes of CIP with 5.45×10^{-3} mol/L pyridine or 4-cyanopyridine and 1.0×10^{-2} mol/L H_2O_2 at 45 °C ($[Co^{III}(opba)]^{-}$-Py-MWCNTs = 0.2 g/L, pH 8.2 (0.01 M borate buffer)).

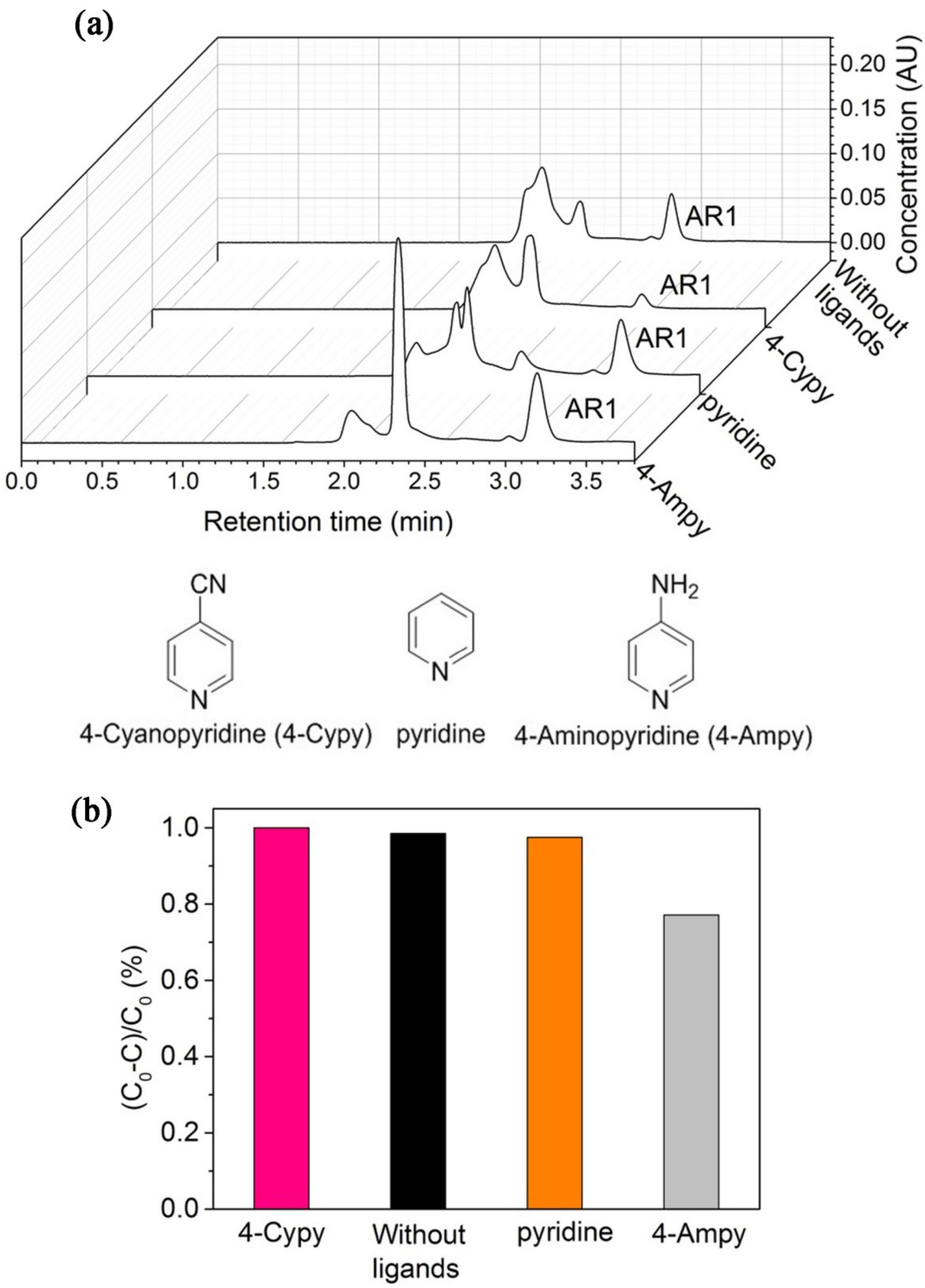

Figure S15. Oxidation of AR1 with different fifth ligands (50 folds as the concentration of $[Co^{III}(opba)]^-$) in 5 min (a) and 30 min (b) with 4.0×10^{-3} mol/L H_2O_2 ($[Co^{III}(opba)]^-$ =1.09×10^{-4} mol/L, T = 25 °C, pH 9.0 (0.01 M borate buffer)).

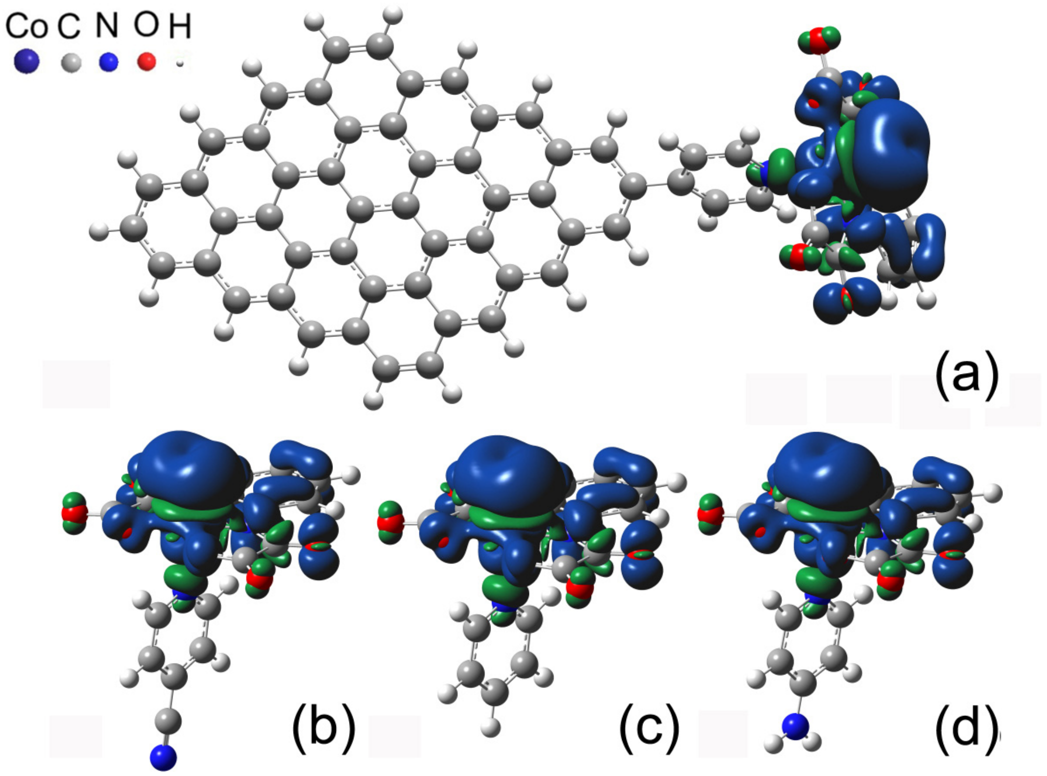

Figure S16. Electron spin population of [•O=Co^{IV}(opba)]$^-$-Py-MWCNTs (a), 4-Cypy-[•O=Co^{IV}(opba)]$^-$ (b), Py-[•O=Co^{IV}(opba)]$^-$ (c) and 4-Ampy-[•O=Co^{IV}(opba)]$^-$ (d) calculated by DFT structures (S=1).

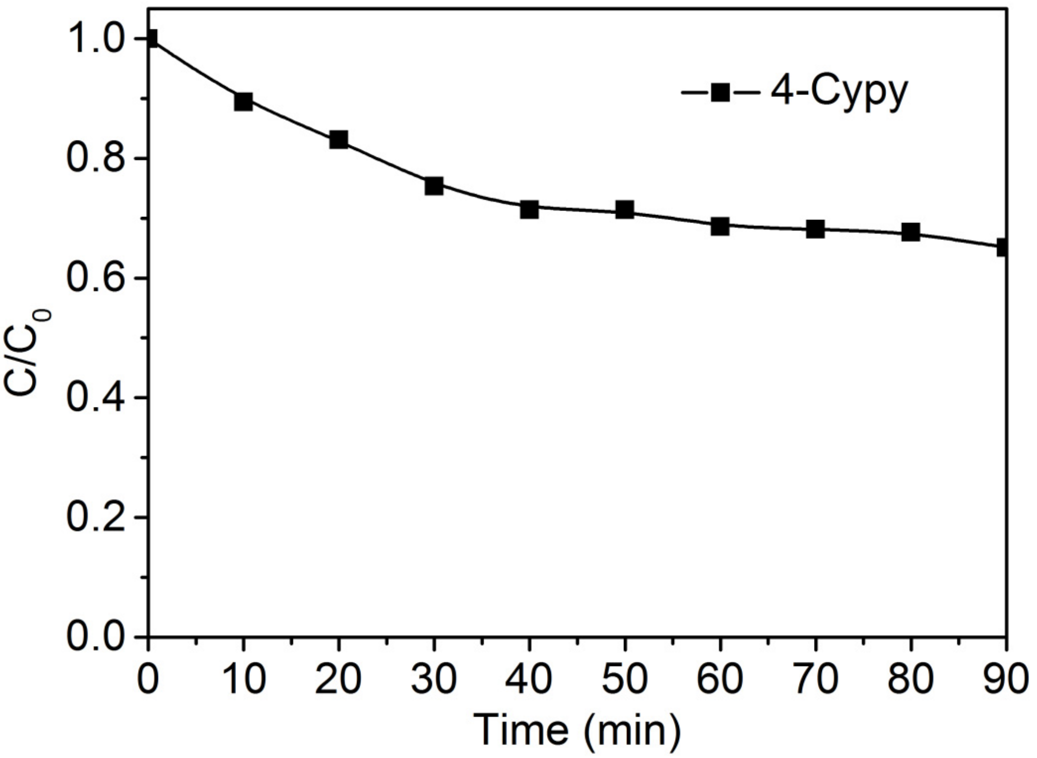

Figure S17. Concentration changes of 4-cyanopyridine in degradation of CIP with 1.0×10^{-2} mol/L H_2O_2 at 45 °C ([Co^{III}(opba)]$^-$-Py-MWCNTs = 0.2 g/L, pH 8.2 (0.01 M borate buffer)).

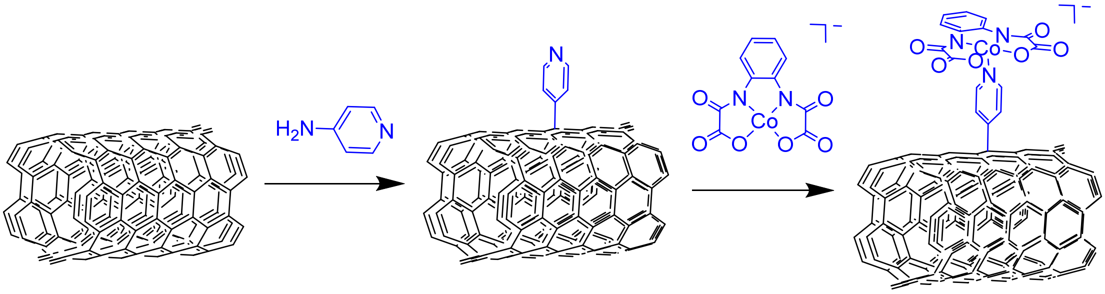

Scheme S1. The preparation of [Co^{III}(opba)]$^{-}$-Py-MWCNTs

Table S1. The total electron energy (a.u.) of 4-Cypy-[•O=Co^{IV}(opba)]$^{-}$, Py-[•O=Co^{IV}(opba)]$^{-}$, 4-Ampy-[•O=Co^{IV}(opba)]$^{-}$ and [•O=Co^{IV}(opba)]$^{-}$-Py-MWCNTs.

**Total energy**	**4-Cypy-[•O=Co^{IV}(opba)]$^{-}$**	**Py-[•O=Co^{IV}(opba)]$^{-}$**	**4-Ampy-[•O=Co^{IV}(opba)]$^{-}$**	**[•O=Co^{IV}(opba)]$^{-}$-Py-MWCNTs**
S=0	-2742.41895793	-2650.20916689	-2705.56804186	-4488.78145424
S=1	-2742.45388633	-2650.24468037	-2705.58996607	-4488.81688322
S=2	-2742.43286901	-2650.22315649	-2705.56804186	-4488.81227805

Table S2. The Co–O bond length of 4-Cypy-[•O=Co^{IV}(opba)]$^{-}$, Py-[•O=Co^{IV}(opba)]$^{-}$, 4-Ampy-[•O=Co^{IV}(opba)]$^{-}$ and MWCNTs-Py-[•O=Co^{IV}(opba)]$^{-}$ calculated by DFT (S=1).

**4-Cypy-[•O=Co^{IV}(opba)]$^{-}$ (Å)**	**Py-[•O=Co^{IV}(opba)]$^{-}$ (Å)**	**4-Ampy-[•O=Co^{IV}(opba)]$^{-}$ (Å)**	**MWCNTs-Py-[•O=Co^{IV}(opba)]$^{-}$ (Å)**
1.67904	1.68345	1.68693	1.68293

Table S3. The optimized structure of MWCNTs-Py-[•O=Co^{IV}(opba)]$^{-}$ calculated by DFT (S=1).

Center Number	Atomic Number	Coordinates (Angstroms)		
		X	Y	Z
1	27	8.725933	0.673146	0.59928
2	8	10.349449	0.995986	0.902979

Table S4. The optimized structure of 4-Cypy-[•O=Co^{IV}(opba)]$^{-}$ calculated by DFT (S=1).

Center Number	Atomic Number	Coordinates (Angstroms)		
		X	Y	Z
1	27	-0.39918	0.000471	-1.18068
2	8	-1.64527	0.000459	-2.30603

Table S5. The optimized structure of 4-Ampy-[•O=Co^{IV}(opba)]$^{-}$ calculated by DFT (S=1).

Center Number	Atomic Number	Coordinates (Angstroms)		
		X	Y	Z
1	27	-0.091089	0.001116	-1.152848
2	8	-1.152636	0.000681	-2.463899

Table S6. The optimized structure of Py-[•O=Co^{IV}(opba)]$^{-}$ calculated by DFT (S=1).

Center Number	Atomic Number	Coordinates (Angstroms)		
		X	Y	Z
1	27	0.362106	0.000075	-0.97484
2	8	-0.3298	0.000083	-2.50953

Table S7. Mulliken charges and spin densities of [•O=Co^{IV}(opba)]$^{-}$-Py-MWCNTs (S=1).

Center Number	Atomic Number	charge density	spin density
1	C	-0.010406	-0.00004
2	C	-0.032497	0.00005
3	C	-0.017591	-0.000039
4	C	-0.017591	0.00005
5	C	-0.010304	-0.000039
6	C	0.005949	0.000029
7	C	-0.057344	0.000042
8	C	0.118086	-0.000062
9	C	-0.253400	0.000115
10	C	0.165112	-0.000062
11	C	0.165030	-0.000063
12	C	-0.253333	0.000117
13	C	0.117980	-0.000063
14	C	-0.182888	0.000113
15	C	-0.122168	-0.00006
16	C	-0.182896	0.000113
17	C	-0.045763	0.000052
18	C	0.140688	-0.00005
19	C	-0.263597	0.000072
20	C	-0.042717	-0.000054
21	C	0.138560	0.000043
22	C	-0.144512	-0.000032
23	C	-0.146157	0.000031
24	C	-0.263664	0.00007
25	C	0.140749	-0.000049
26	C	-0.045884	0.000051
27	C	-0.146171	0.00003
28	C	-0.144589	-0.000031
29	C	0.138630	0.000041
30	C	-0.042693	-0.000053
31	C	0.012734	-0.000015
32	C	-0.015470	0.000025
33	C	-0.015521	0.000025
34	C	-0.024697	-0.000015
35	C	-0.023431	-0.000001
36	C	-0.024657	-0.000016
37	C	-0.258497	-0.000068
38	C	0.159685	0.00003
39	C	-0.243634	-0.00007
40	C	0.113466	-0.000027
41	C	-0.046119	0.000067
42	C	-0.203611	0.000119
43	C	0.048954	-0.00022
44	C	-0.203040	0.000103
45	C	0.112970	-0.000026
46	C	-0.243395	-0.000073
47	C	0.159457	0.000033
48	C	-0.258340	-0.00007
49	H	0.129142	-0.000004

50	H	0.129189	-0.000004
51	H	0.123764	-0.000005
52	H	0.120557	0.000002
53	H	0.123698	-0.000005
54	H	0.131523	-0.000002
55	H	0.133450	0.000001
56	H	0.129817	-0.000001
57	H	0.131465	-0.000002
58	H	0.129696	-0.000001
59	H	0.133016	0.000001
60	H	0.139077	0.000002
61	H	0.142708	0.000002
62	H	0.149923	-0.000004
63	H	0.153086	-0.000006
64	H	0.144265	0.000002
65	H	0.139884	0.000002
66	C	0.142504	0.00095
67	C	-0.141634	-0.001684
68	C	0.023038	0.001453
69	C	-0.139290	-0.001416
70	C	0.109542	0.003314
71	N	-0.559980	-0.017008
72	H	0.237306	-0.000485
73	H	0.148949	-0.000083
74	H	0.146144	-0.000082
75	H	0.197209	-0.000605
76	C	-0.145487	0.011482
77	C	-0.145275	0.009731
78	C	-0.107505	0.004151
79	C	0.267528	0.006603
80	C	0.265893	0.0085
81	C	-0.109080	0.002414
82	H	0.102279	-0.000642
83	H	0.102523	-0.000568
84	H	0.170026	-0.000401
85	H	0.169578	-0.000333
86	N	-0.768425	0.034409
87	N	-0.771092	0.03193
88	C	0.445521	-0.007604
89	O	-0.459330	0.031778
90	C	0.508556	-0.001704
91	O	-0.439353	-0.004614
92	C	0.444239	-0.007147
93	O	-0.461064	0.029179
94	C	0.509883	-0.001751
95	O	-0.438540	-0.00461
96	Co	1.111821	0.707616
97	O	-0.576723	0.017356
98	O	-0.581035	0.017336
99	O	-0.279417	1.132433

Table S8. Mulliken charges and spin densities of 4-Cypy-[•O=Co^{IV}(opba)]$^{-}$ (S=1).

Center Number	Atomic Number	charge density	spin density
1	C	0.147969	0.000758
2	C	-0.093355	-0.001486
3	C	0.098629	0.001305
4	C	-0.091764	-0.001497
5	C	0.112517	0.003215
6	N	-0.548901	-0.015801
7	H	0.246695	-0.000435
8	H	0.173840	-0.000061
9	H	0.170783	-0.000062
10	H	0.206027	-0.000578
11	C	-0.144893	0.011348
12	C	-0.144894	0.011309
13	C	-0.107457	0.002884
14	C	0.265685	0.008594
15	C	0.265671	0.008637
16	C	-0.107475	0.002845
17	H	0.105245	-0.000636
18	H	0.105244	-0.000634
19	H	0.171556	-0.000359
20	H	0.171554	-0.000357
21	N	-0.772022	0.034926
22	N	-0.772061	0.03487
23	C	0.445417	-0.007721
24	O	-0.455602	0.031063
25	C	0.508639	-0.001662
26	O	-0.433449	-0.004564
27	C	0.445398	-0.007712
28	O	-0.455627	0.030999
29	C	0.508657	-0.001664
30	O	-0.433446	-0.004561
31	Co	1.114214	0.707663
32	O	-0.581150	0.018248
33	O	-0.581208	0.018242
34	O	-0.277767	1.122794
35	C	-0.029891	-0.000367
36	N	-0.232778	0.00046

Table S9. Mulliken charges and spin densities of Py-[•O=Co^{IV}(opba)]$^{-}$ (S=1).

Center Number	Atomic Number	charge density	spin density
1	C	0.147798	0.0011
2	C	-0.129865	-0.001791
3	C	-0.110360	0.001564
4	C	-0.127516	-0.001442
5	C	0.113480	0.003417
6	N	-0.561944	-0.017457
7	H	0.236509	-0.000502
8	H	0.145228	-0.000097
9	H	0.142017	-0.000088
10	H	0.196411	-0.000631
11	C	-0.145441	0.010497
12	C	-0.145442	0.010477
13	C	-0.108468	0.003413
14	C	0.266936	0.007316
15	C	0.266928	0.007338
16	C	-0.108477	0.003394
17	H	0.101362	-0.000601
18	H	0.101362	-0.0006
19	H	0.169346	-0.00037
20	H	0.169346	-0.000369
21	N	-0.769449	0.03276
22	N	-0.769467	0.032733
23	C	0.445044	-0.007282
24	O	-0.461214	0.030298
25	C	0.509819	-0.001718
26	O	-0.439839	-0.00463
27	C	0.445033	-0.007277
28	O	-0.461227	0.030264
29	C	0.509830	-0.001719
30	O	-0.439839	-0.004629
31	Co	1.112356	0.709921
32	O	-0.578828	0.017096
33	O	-0.578860	0.017094
34	O	-0.280702	1.132603
35	H	0.138133	-0.000082

Table S10. Mulliken charges and spin densities of 4-Ampy-[•O=Co^{IV}(opba)]$^{-}$ (S=1).

Center Number	Atomic Number	charge density	spin density
1	C	0.139653	0.000816
2	C	-0.148737	-0.001698
3	C	0.334702	0.001276
4	C	-0.147139	-0.000903
5	C	0.107916	0.00325
6	N	-0.581414	-0.018042
7	H	0.229054	-0.000507
8	H	0.128524	-0.000095
9	H	0.125508	-0.000111
10	H	0.189378	-0.000617
11	C	-0.145964	0.009748
12	C	-0.145973	0.009671
13	C	-0.109246	0.003779
14	C	0.267352	0.006221
15	C	0.267316	0.006305
16	C	-0.109278	0.003704
17	H	0.098851	-0.000568
18	H	0.098851	-0.000565
19	H	0.167795	-0.000376
20	H	0.167796	-0.000373
21	N	-0.767269	0.030715
22	N	-0.767307	0.030606
23	C	0.444597	-0.006916
24	O	-0.465166	0.029608
25	C	0.510327	-0.001777
26	O	-0.444531	-0.00466
27	C	0.444552	-0.006898
28	O	-0.465218	0.029481
29	C	0.510341	-0.001778
30	O	-0.444537	-0.004653
31	Co	1.110610	0.711017
32	O	-0.577096	0.01609
33	O	-0.577175	0.016077
34	O	-0.282443	1.141578
35	N	-0.804320	0.000673
36	H	0.319111	-0.000034
37	H	0.320575	-0.000046

Table S11. Oxidative intermediates of AR1 in the presence of $[Co^{III}(opba)]^-$-Py-MWCNTs and H_2O_2 examined by UPLC Synapt G2-S HDMS in the negative ion mode after 60 minutes of reaction time.

Intermediate Products	Retention Time (min)	Theoretical Mass (m/z)	Molecular Formula	Measured Accurate Mass (m/z)	mDa
P_1	5.45	400.0603	$C_{18}H_{15}N_3O_6S$	400.0603	0
P_2	6.08	438.0607	$C_{17}H_{17}N_3O_9S$	438.0610	0.3
P_3	5.03	316.0127	$C_{11}H_{11}NO_8S$	316.0129	0.2
P_4	3.35	286.0021	$C_{10}H_9NO_7S$	286.0029	0.8
P_5	4.2	270.0072	$C_{10}H_9NO_6S$	270.0073	0.1
P_6	5.32	165.0188	$C_8H_6O_4$	165.0175	-1.3
P_7	8.15	137.0239	$C_7H_6O_3$	137.0233	-0.6
P_8	0.71	122.0368	$C_7H_6O_2$	122.0318	-5
P_9	0.91	191.0192	$C_6H_8O_7$	191.0184	-0.8
P_{10}	0.72	145.0501	$C_6H_{10}O_4$	145.0538	-3.7
P_{11}	0.99	147.0293	$C_5H_8O_5$	147.0266	-2.7
P_{12}	0.72	116.0110	$C_4H_4O_4$	116.0078	-3.2
P_{13}	2.32	117.0188	$C_4H_6O_4$	117.0190	0.2
P_{14}	0.89	149.0086	$C_4H_6O_6$	149.0079	-0.7
P_{15}	1.12	115.0031	$C_3H_4O_4$	115.0026	-0.5

References

1. Wan, K.; Yu, Z.-p.; Li, X.-h.; Liu, M.-y.; Yang, G.; Piao, J.-h.; Liang, Z.-x., PH effect on electrochemistry of nitrogen-doped carbon catalyst for oxygen reduction reaction. *ACS Catal.* **2015,** 5, (7), 4325-4332.
2. Wang, Y.; Liu, J.; Wang, P.; Werth, C. J.; Strathmann, T. J., Palladium nanoparticles encapsulated in core–shell silica: A structured hydrogenation catalyst with enhanced activity for reduction of oxyanion water pollutants. *ACS Catal.* **2014,** 4, (10), 3551-3559.
3. Ojima, H.; Nonoyama, K., Copper (II) complexes with N, N′-bis (alkylaminoalkyl)-oxamides and related ligands. *Coord. Chem. Rev.* **1988,** 92, 85-111.
4. Ruiz, R.; Faus, J.; Lloret, F.; Julve, M.; Journaux, Y., Coordination chemistry of N, N′-bis (coordinating group substituted) oxamides: a rational design of nuclearity tailored polynuclear complexes. *Coord. Chem. Rev.* **1999,** 193, 1069-1117.
5. Lu, W.; Li, N.; Bao, S.; Chen, W.; Yao, Y., The coupling of metallophthalocyanine with carbon nanotubes to produce a nanomaterial-based catalyst for reaction-controlled interfacial catalysis. *Carbon* **2011,** 49, (5), 1699-1709.
6. Kasiri, M.; Aleboyeh, H.; Aleboyeh, A., Degradation of Acid Blue 74 using Fe-ZSM5 zeolite as a heterogeneous photo-Fenton catalyst. *Appl. Catal. B: Environ.* **2008,** 84, (1), 9-15.
7. Dean, D.; Davis, B.; Jessop, P. G., The effect of temperature, catalyst and sterics on the rate of N-heterocycle dehydrogenation for hydrogen storage. *New J. Chem.* **2011,** 35, (2), 417-422.

8. Chen, J.; Zhu, L., Heterogeneous UV-Fenton catalytic degradation of dyestuff in water with hydroxyl-Fe pillared bentonite. *Catal. Today* **2007,** 126, (3), 463-470.
